# Temporal Shift of Circadian-Mediated Gene Expression and Carbon Fixation Contributes to Biomass Heterosis in Maize Hybrids

**DOI:** 10.1371/journal.pgen.1006197

**Published:** 2016-07-28

**Authors:** Dae Kwan Ko, Dominica Rohozinski, Qingxin Song, Samuel H. Taylor, Thomas E. Juenger, Frank G. Harmon, Z. Jeffrey Chen

**Affiliations:** 1 Department of Molecular Biosciences, The University of Texas at Austin, Austin, Texas, United States of America; 2 Plant Gene Expression Center, USDA-ARS, Albany, California, United States of America; 3 Department of Plant & Microbial Biology, University of California, Berkeley, California, United States of America; 4 Department of Integrative Biology, Center for Computational Biology and Bioinformatics, and Institute for Cellular and Molecular Biology, The University of Texas at Austin, Austin, Texas, United States of America; Stanford University School of Medicine, UNITED STATES

## Abstract

Heterosis has been widely used in agriculture, but the molecular mechanism for this remains largely elusive. In *Arabidopsis* hybrids and allopolyploids, increased photosynthetic and metabolic activities are linked to altered expression of circadian clock regulators, including *CIRCADIAN CLOCK ASSOCIATED1* (*CCA1*). It is unknown whether a similar mechanism mediates heterosis in maize hybrids. Here we report that higher levels of carbon fixation and starch accumulation in the maize hybrids are associated with altered temporal gene expression. Two maize *CCA1* homologs, *ZmCCA1a* and *ZmCCA1b*, are diurnally up-regulated in the hybrids. Expressing *ZmCCA1* complements the *cca1* mutant phenotype in *Arabidopsis*, and overexpressing *ZmCCA1b* disrupts circadian rhythms and biomass heterosis. Furthermore, overexpressing *ZmCCA1b* in maize reduced chlorophyll content and plant height. Reduced height stems from reduced node elongation but not total node number in both greenhouse and field conditions. Phenotypes are less severe in the field than in the greenhouse, suggesting that enhanced light and/or metabolic activities in the field can compensate for altered circadian regulation in growth vigor. Chromatin immunoprecipitation-sequencing (ChIP-seq) analysis reveals a temporal shift of ZmCCA1-binding targets to the early morning in the hybrids, suggesting that activation of morning-phased genes in the hybrids promotes photosynthesis and growth vigor. This temporal shift of ZmCCA1-binding targets correlated with nonadditive and additive gene expression in early and late stages of seedling development. These results could guide breeding better hybrid crops to meet the growing demand in food and bioenergy.

## Author Summary

All corn in the USA is grown as hybrids, which grow more vigorously and produce higher yield than their parents, a phenomenon known as heterosis. The molecular basis for heterosis remains elusive. Heterosis is predicted to arise from allelic interactions between parental genomes, leading to altered regulatory networks that promote the growth and fitness of hybrids. One such regulator is the circadian clock, which is functionally conserved in *Arabidopsis* and maize. Disrupting corn *CCA1* expression reduces growth vigor. In corn hybrids, CCA1 proteins target thousands of output genes early in the morning, as if the hybrids wake up early to promote photosynthesis, starch metabolism and biomass accumulation. This early acting mechanism could guide breeding and selection of high-yield hybrids.

## Introduction

A filial F1 hybrid often outperforms inbred parents in growth and fitness, a phenomenon known as heterosis or hybrid vigor. Since early twentieth century, heterosis has been applied in breeding and production of maize and many other crops, vegetables, and some animals [1–3]. However, the molecular bases for heterosis are poorly understood. Traditional “dominance” and “overdominance” models cannot explain the complexity of heterosis. It is suggested that these allelic models should be updated to account for gene expression and regulatory networks that are often altered in hybrids relative to their parents [3–5]. Notably, gene expression changes are dynamic in crop hybrids. For example, maize F1 hybrids exhibit additive expression, non-additive expression [6, 7], and all possible modes of gene expression [8]. These different observations of expression changes could be associated with a few major regulatory genes that cause a cascade of effects on downstream metabolic and physiological pathways, leading to heterosis [5].

Consistent with this concept, recent studies have discovered a link between altered circadian clock regulation and increased levels of photosynthetic and metabolic activities and biomass in *Arabidopsis* allotetraploids (interspecific hybrids) [9] and *Arabidopsis thaliana* intraspecific hybrids [10, 11]. These results collectively indicate that the expression of central circadian clock genes is epigenetically altered in hybrids, which in turn increases expression levels of downstream genes involved in energy and metabolic pathways, promoting carbohydrate metabolism during the day and night. The more starch accumulates during the day; the more starch can be degraded at night to stimulate growth, leading to biomass heterosis [5].

In *A*. *thaliana*, the circadian clock consists of central oscillator components CIRCADIAN CLOCK-ASSOCIATED 1 (CCA1) and LATE ELONGATED HYPOCOTYL (LHY) and their reciprocal regulator TIMING OF CAB EXPRESSION 1 (TOC1), also known as PSEUDORESPONSE REGULATOR1 (PRR1) [12, 13]. When the endogenous clock matches the external diurnal cycle, CO_2_ fixation, photosynthetic activities, and fitness are increased [14]. Disrupting clock function causes decreased growth and fitness. For example, a *CCA1* overexpression mutant (*CCA1-OX*) lacks circadian rhythms and displays reduced photosynthesis and fitness [15]. The double mutant *cca1 lhy* accumulates less starch and is unable to properly set the rate of starch degradation to match the length of night [16]. An important mechanism by which the clock improve fitness is temporal regulation of energetically costly activities, also known as gating [17]. Gating is apparent for pathogen responses, temperature responses, growth control, shade avoidance, and phytohormone signaling [18–25]. *CCA1-OX* plants are arrhythmic and largely lack these behaviors.

Maize performs C_4_ photosynthesis, which is anatomically and biochemically distinct from C_3_ photosynthesis in plants like *Arabidopsis* [26]. Photosynthetic activities in maize are subject to diurnal regulation [27]. Sucrose accumulation rates increase during the day, reach a high level at 15:00 hours, and continue to increase until dusk. Starch mobilization occurs beginning at dusk, and all of the starch is depleted by the end of the night. Mobilization of starch at night likely promotes growth, which, after temperature correction, is greatest at night [28]. This diurnal regulation of carbohydrate metabolism is consistent with diurnal gene expression in maize leaves. Approximately 10% of ~13,000 transcripts examined display circadian expression patterns [29]. The majority of cycling genes peak their expression at subjective dawn and dusk, similar to other plant circadian systems. In another study, 23% of expressed transcripts exhibit a diurnal cycling pattern in leaves [30]. By contrast, in developing ears only core circadian clock genes and a handful of other transcripts are diurnally regulated. This suggests tissue-specific circadian regulation in maize, as observed in *Arabidopsis* [10, 31]. The circadian effect on biomass heterosis is established early during embryo development [10]. Furthermore, developmental shifts in gene expression can also influence biomass accumulation. In sorghum, SbPRR37 activates expression of several downstream genes that repress flowering in long days [32]. *SbPRR37* expression is dependent on light and regulated by the circadian clock. In short days, *SbPRR37* is not expressed during the evening-phase, allowing sorghum to flower. This suggests an integration of circadian clock with flowering time that alters biomass production.

Quantitative trait loci (QTLs) for key agronomic traits in crop plants have been associated with likely clock-associated genes [33]. For example, yield-related QTLs are mapped to clock-related and light signaling genes in the super-hybrid rice [34]. Flowering QTLs are associated with circadian clock regulators, *GIGANTEA* in soybean [35] and *CONSTANS* in sorghum [36]. A recent study finds that the allelic variation of clock loci in tomato varieties is linked to their domestication [37]. In maize, agronomic traits are controlled by many small-effect QTLs [38], and several clock regulator homologs are identified as *a priori* candidate genes among genome-wide association QTLs [39]. These studies support the notion that the circadian clock mediates flowering, growth, and heterosis in crop plants including maize as in *Arabidopsis* [40].

However, the effect of the circadian clock on biomass production in maize hybrids and their inbred lines is largely unknown. Here we examined biomass accumulation in maize hybrids and their inbred parents, and investigated the function of maize *CCA1* homologs (*ZmCCA1a* and *ZmCCA1b*). Cloned *ZmCCA1* coding sequences were used to test their functions in both *Arabidopsis* and maize. Recombinant ZmCCA1 proteins were used to study their binding affinity to promoter fragments *in vitro*, and polyclonal antibodies against ZmCCA1 were used to investigate ZmCCA1-binding targets and genes *in planta*. The results indicate altered temporal binding activities of ZmCCA1s in hybrids relative to inbred parents, associated with increased expression levels of carbon fixation genes, as well as increased rates of carbon fixation and biomass accumulation. The data suggest a molecular mechanism that reprograms circadian-regulatory networks in maize hybrids, promoting growth vigor.

## Results

### Early establishment of growth vigor in maize hybrids

**Fig 1:**
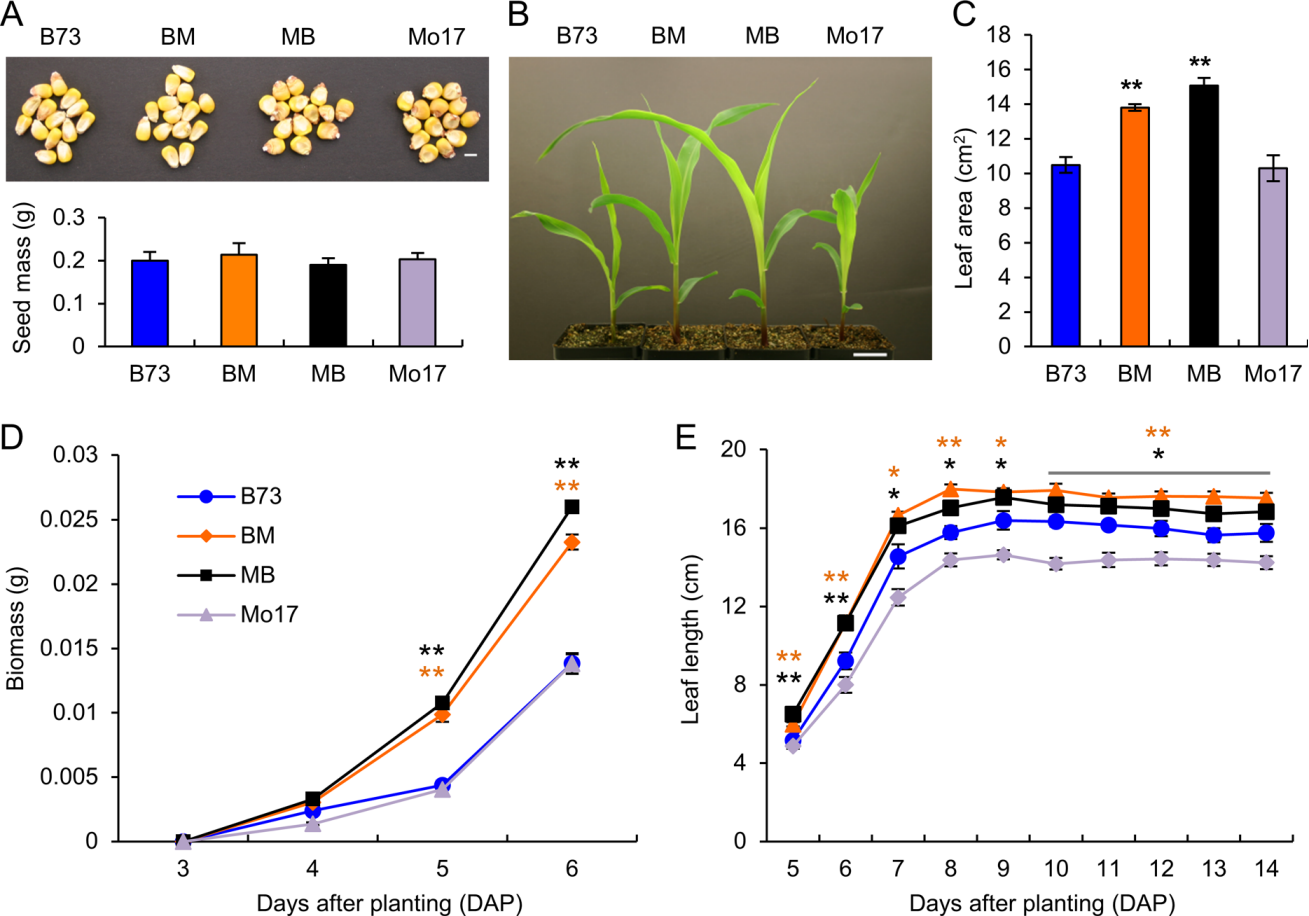
Growth heterosis starts at the early seedling stage. (A) Seed morphology (upper panel) and weight (lower panel, means ± SEM) in maize inbred lines (B73 and Mo17) and their reciprocal F1 hybrids (B73XMo17, BM and Mo17XB73, MB). Scale bar = 10 mm. (B) Seedling phenotypes showing growth vigor in maize F1 hybrids. Scale bar = 10 mm. (C) Leaf area of F1 hybrids relative to the inbreds (means ± SEM, n = 10). (D) Growth curve of biomass in the maize inbreds and F1 hybrids (means ± SEM, n = 6). (E) Growth curves of leaf length in the maize inbreds and F1 hybrids (means ± SEM, n = 5). Heterosis for biomass and leaf length was observed at 5 DAP in the hybrids. Note that the earliest time to measure biomass was 3 DAP when young leaves emerged. Significant difference between hybrids and the best parent was calculated using Student’s t-test, *p-value < 0.05 and **p-value < 0.01.

Seedling growth vigor was reported to correlate with larger seeds in F1 hybrids [41]. This hypothesis was not supported by seed size analysis: seed weight was similar in maize inbred lines (B73 and Mo17) and their reciprocal F1 hybrids (B73XMo17, BM, and Mo17XB73, MB) (by convention, the female parent is listed first in a genetic cross) (Fig 1A). In spite of similar seed size, seedling growth heterosis occurred soon after germination (Fig 1B), and leaf areas were significantly greater in the F1 hybrids than in the parents (Fig 1C). F1 hybrids showed biomass heterosis 5 days after planting (5 DAP) (Fig 1D), and leaf length of the hybrids was significantly greater than the best parent from the emergence of the first true leaf 5 DAP until 14 DAP (Fig 1E). Biomass and plant height heterosis was significantly different between the reciprocal crosses, and vigor was higher when Mo17 was used as the maternal parent. However, this difference in height decreased and was no longer obvious after two weeks (Fig 1D and S1 Fig). These data suggest early establishment of seedling growth heterosis and subsequent maintenance of this heterosis during seedling development.

**Fig 2:**
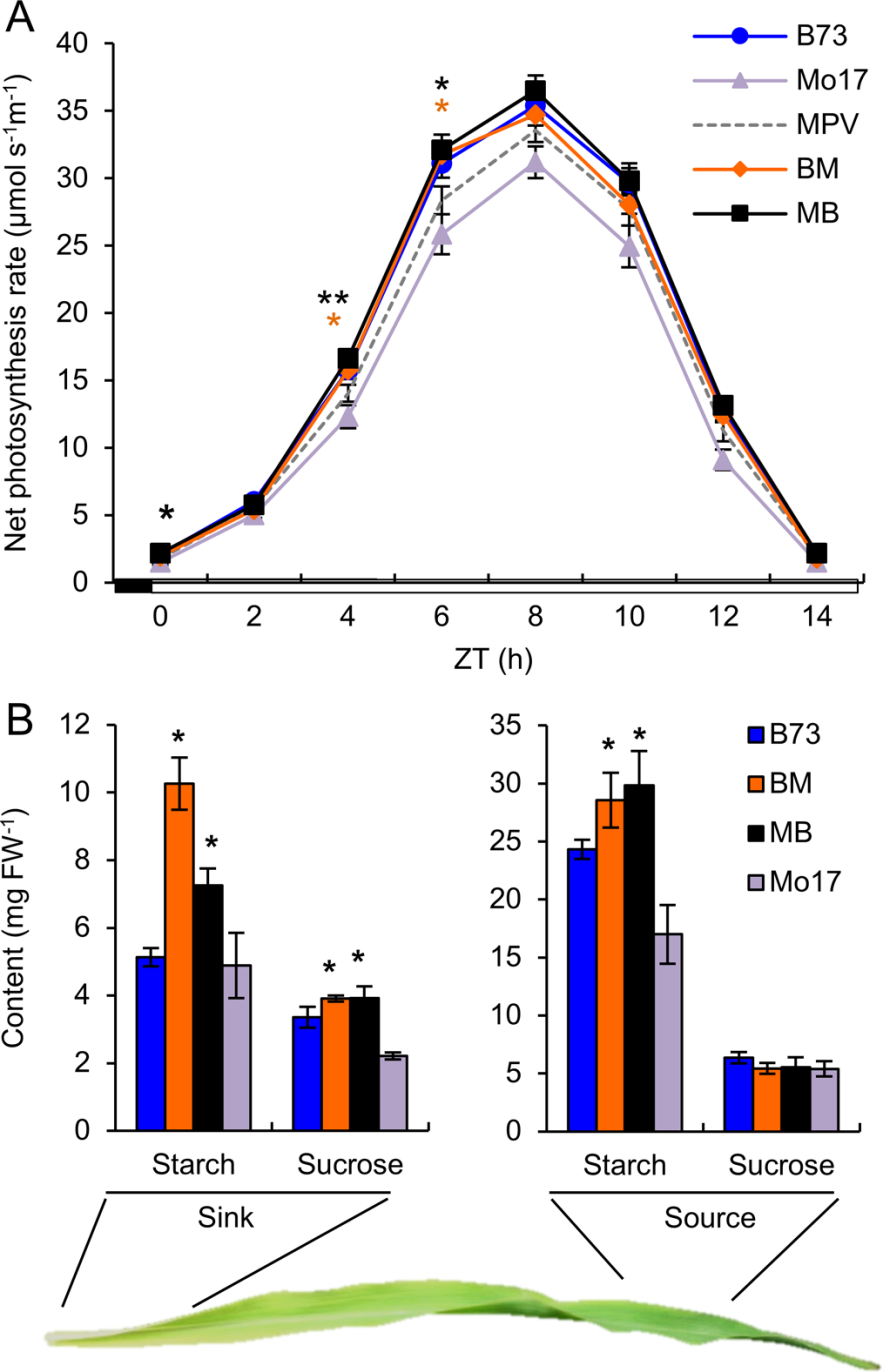
Increased capacity of carbon fixation in the F1 hybrids relative to the inbreds. (A) Diurnal rhythms of net photosynthesis rate in the maize inbreds and hybrids (means ± SEM, n = 9). (B) Starch and sucrose accumulation in sink and source regions of a mature leaf (means ± SEM, n = 3) at ZT14. FW, fresh weight. Significant difference between hybrids and MPV was calculated using Student’s t-test, *p-value < 0.05 and **p-value < 0.01.

Seedling growth rates were associated with increased levels of photosynthetic activities including CO_2_ assimilation, which is under diurnal regulation as observed in maize inbred lines [27]. Positive net CO_2_ assimilation increased from zeitgeber time 0 (ZT0 = dawn), peaked at ZT8, declined through the remaining light period, and ceased at ZT14 (Fig 2A). Notably, the amplitude of CO_2_ assimilation significantly increased in both hybrids relative to the mid-parent value (MPV), especially during the morning phases. This increase in photosynthetic rate of the hybrids should be related to the expected effect of greater leaf area on whole-plant carbon gain relative to the inbreds.

The increased photosynthetic activities promote starch synthesis and mobilization. In a maize leaf, the middle-tip region represents a source where carbohydrates are synthesized, whereas the base region represents a sink where carbohydrates are stored, utilized in growth, and mobilized [26, 42]. In the source region, starch amount was significantly more in the hybrids than the MPV, but the sucrose content was similar (Fig 2B). In the sink region, both starch and sucrose accumulated significantly more in the hybrids than the MPV. Little photosynthetic activity is present in the sink region of a maize leaf [43]; thus starch accumulation is likely caused by the increased CO_2_ assimilation in the source region during the day, with carbohydrates being mobilized to the sink region at night. More sucrose synthesis and mobilization in the hybrids provides more carbohydrates for seedling growth, suggesting a role for diurnal regulation of carbon metabolism in growth heterosis.

### Maize CCA1 homologs exhibit diurnal expression patterns and binding activities

**Fig 3:**
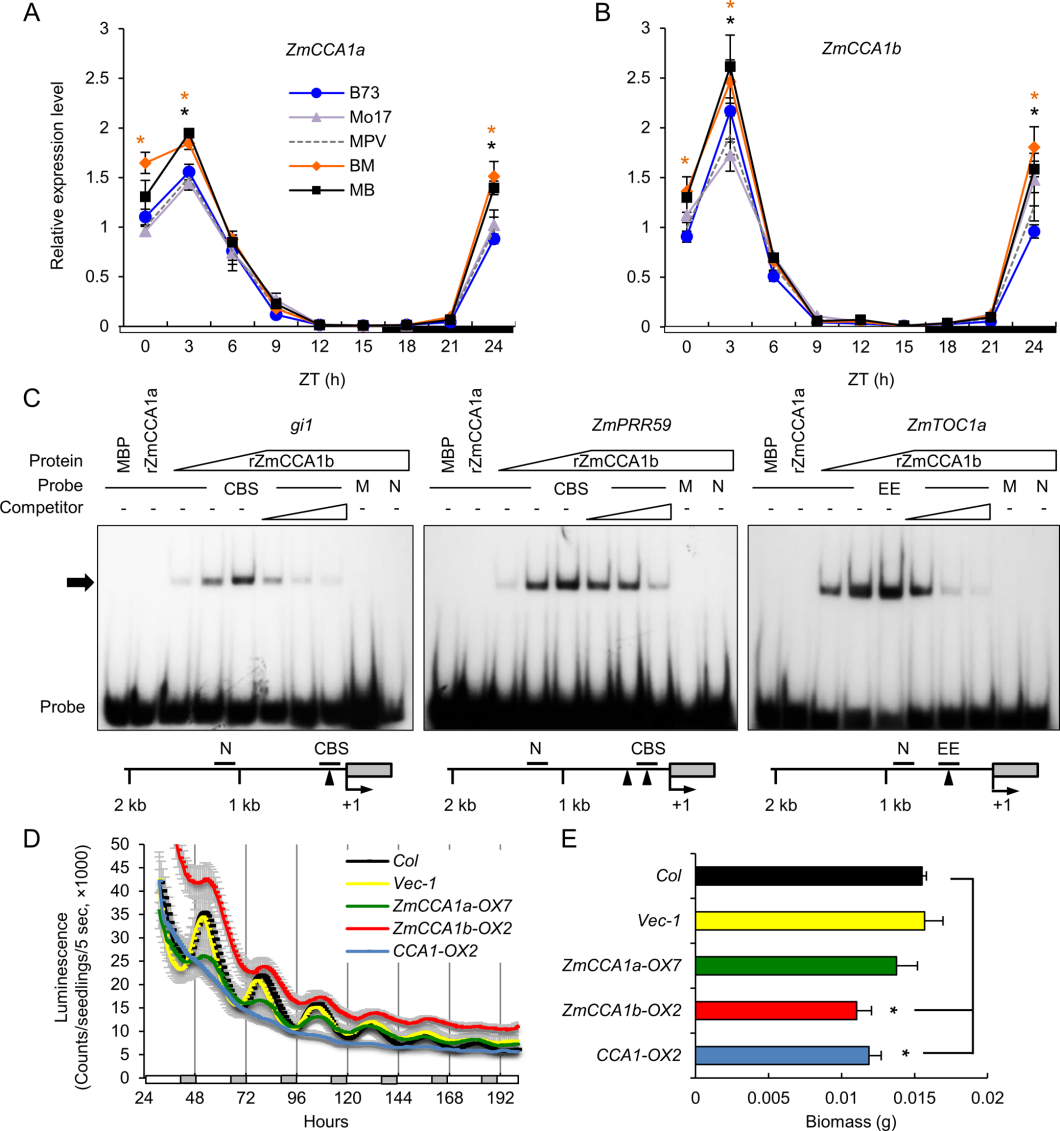
Molecular characterization of maize CCA1 homologs. (A and B) Relative expression levels (means ± SEM, n = 3) of *ZmCCA1a* (A) and *ZmCCA1b* (B) every 3 hours in a 24-hour period (light/dark cycle is shown below the histogram). Significant difference between hybrids and MPV was calculated using Student’s t-test, *p-value < 0.05. The relative expression level in MPV at ZT0 was set to 1. (C) Binding of recombinant ZmCCA1b (rZmCCA1b) to promoters of putative maize clock genes *in vitro*. Radioisotope-labeled DNA probes (endogenous promoter fragments) were incubated in the presence of MBP (1 pmol), rZmCCA1a (1 pmol), and rZmCCA1b (0.5, 1 and 2 pmol). Shifted protein-DNA complexes are indicated by the arrow. Competitors: 25X, 50X and 100X molar excess of unlabeled prompter DNA. M: DNA in which EE or CBS site was mutated; N: no EE or CBS site in the DNA fragment. Location of probes for each gene is shown below the gel image. Arrowheads represent EE or CBS site. Numbers are relative to the transcription start site (+1) and 5” UTR (grey box). (D) *CAB2*:*LUC* activity rhythms in wild-type (Col-0), *Vec-1*, *ZmCCA1a-OX7*, *ZmCCA1b-OX2* and *CCA1-OX2* under constant light (means ± SEM, n = 12–16). White and grey bars represent the subjective day and night, respectively. T2 plants were used in the analysis. (E) Dry aerial biomass of wild-type (Col-0), *Vec-1*, *ZmCCA1a-OX7*, *ZmCCA1b-OX2* and *CCA1-OX2*. Dry biomass (grams) was measured 35 DAP in the diurnal conditions (means ± SEM, n = 10).

Photosynthetic activities and starch metabolism are diurnally regulated in maize [27, 28] as in *Arabidopsis* diploids, hybrids and allopolyploids [5], suggesting a role for the circadian clock in promoting seedling growth in maize. To test this, we investigated *CCA1* homologs and their functions in maize. Being an ancient tetraploid [44, 45], maize has paralogous duplicates of many clock gene homologs [30]. The two homologs, designated *ZmCCA1a* (*ZM2G474769*) and *ZmCCA1b* (*ZM2G014902*) and located on chromosomes 10 and 4, respectively [30], are closely related to *CCA1* and *LHY* in *Arabidopsis* (S2A Fig). In the maize gene model (B73 RefGen_v2), *ZmCCA1a* is a truncated version; in the ~20-kb upstream, there is another homologous gene (*ZM2G175265*) that encodes a MYB DNA-binding domain protein. The short transcript (*ZM2G474769*, *ZmCCA1a*) could be a splicing variant and was used for expression and other analyses in this study. In *Arabidopsis*, CCA1 lacking the MYB DNA-binding domain acts as a dominant negative factor to fine-tune the period length [46]. The two maize genes exhibit diurnal expression patterns, although *ZmCCA1b* was expressed at higher levels than *ZmCCA1a* [30], in most of 17 maize tissues examined [47] (S2C Fig), indicating that *ZmCCA1b* may play a larger role than this truncated *ZmCCA1a*. Expression of *ZmCCA1a* and *ZmCCA1b* was diurnally regulated and peaked at ZT3 (Fig 3A), whereas *GIGANTEA1* (*gi1*), *ZmPRR59*, and *ZmTOC1a* [30, 33], peaked at ZT12 (S6A–S6C Fig). Interestingly, all clock genes analyzed were nonadditively expressed (deviated from MPV) in the hybrids at time-points that coincided with nonadditive photosynthetic and metabolic activities during the day (Fig 2). In the hybrids, both *ZmCCA1a* and *ZmCCA1b* were up-regulated in the morning phase, while *gi1* and *ZmPRR59*, a *PRR5* homolog in *Arabidopsis*, were up-regulated at ZT9. However, *ZmTOC1a* was down-regulated in the middle of the day and at night. These data indicate altered expression of circadian clock genes in the maize hybrids as in *Arabidopsis* allotetraploids [9] and intraspecific hybrids [10, 48].

Altered *CCA1* expression may affect abundance of CCA1 protein that binds to the evening element (EE) or CCA1-binding site (CBS) in the promoters of other clock and output genes [9, 49]. Electrophoretic mobility shift assays (EMSA) showed that ZmCCA1b but not ZmCCA1a (excluding the MYB DNA-binding domain) bound competitively and specifically to the EE and CBS elements of *ZmTOC1a*, *gi1* and *ZmPRR59* promoter fragments *in vitro* (Fig 3C). The binding signal depended on EE or CBS elements as well as the recombinant ZmCCA1b (rZmCCA1b) protein concentration. When the EE or CBS was mutated (M) or no EE or CBS (N) was present in the endogenous promoter fragments, the binding activity of rZmCCA1b was undetectable. As a control, maltose-binding protein (MBP) showed no binding activities. Like the control, rZmCCA1a showed no binding activity. The absence of the MYB DNA-binding domain in truncated ZmCCA1a likely explains undetectable target binding. These data suggest that ZmCCA1b is a functional protein that binds to the promoters of circadian clock genes via *cis*-acting EE and CBS elements.

### Functional conservation of maize circadian clock genes

To test the function of maize CCA1 homologs, we generated *Arabidopsis CCA1*, maize *ZmCCA1a* or *ZmCCA1b* overexpression (OX) lines in the transgenic *A*. *thaliana* (Col-0) plants that also express a luciferase (LUC) reporter driven by the promoter of *CHLOROPHYLL A/B BINDING PROTEIN 2* (*CAB2*) (*CAB2*:*LUC*). These lines designated *CCA1-OX*, *ZmCCA1a-OX*, and *ZmCCA1b-OX*, respectively (Fig 3D). While the wild-type and transgenic control (*Vec-1*) lines showed rhythmic *CAB2*:*LUC* expression, overexpressing *CCA1* abolished *CAB2*:*LUC* expression rhythmicity under constant light (S3A Fig), as previously reported (Wang and Tobin, 1998 Cell). Interestingly, in the *ZmCCA1b-OX* lines the *CAB2*:*LUC* expression rhythmicity was dampened, and the timing of peak expression delayed under the constant light. Overexpressing *ZmCCA1a* in the *ZmCCA1a-OX* lines had a smaller effect on *CAB2*:*LUC* expression rhythmicity, and the expression peak was lower than that in the *ZmCCA1b-OX* lines (Fig 3D and S3A Fig). The weaker *ZmCCA1a-OX* phenotype may be associated with the lack of binding activity in the truncated ZmCCA1a to the EE or CBS of gene promoters (Fig 3C).

As a result of disrupting the clock gene functions, aerial biomass of *CCA1-OX* and *ZmCCA1b-OX* lines was significantly lower than the wild-type or the transgenic control (Fig 3E), consistent with the growth disadvantage in clock gene mutants and overexpression lines [14, 15]. Biomass in the *ZmCCA1a-OX* lines was not significantly different from the controls, suggesting that a full-length clock gene is required for growth vigor.

Expressing *ZmCCA1a* or *ZmCCA1b* under the *Arabidopsis CCA1* promoter partially rescued the early period phenotype of *CAB2*:*LUC* in the *cca1-11* mutant (S3B and S3C Fig). Under constant light, the period of *CAB2*:*LUC* rhythms was shorter in the *cca1-11* mutant (23.9 ± 0.1 h) than in the wild-type (WS) (25.4 ± 0.3 h). Expressing *Arabidopsis CCA1* in the *cca1-11* mutant, as a control, fully rescued the short period phenotype of *CAB2*:*LUC* (25.4 ± 0.1 h). Expressing *CCA1*:*ZmCCA1b* (24.8 ± 0.1 h) or *CCA1*:*ZmCCA1a* (24.2 ± 0.1 h) in the *cca1* mutant also lengthened the period relative to *cca1-11* (23.9 ± 0.1 h). These data suggest that the circadian clock function between *Arabidopsis* and maize *CCA1* homologs is conserved. *ZmCCA1b* complemented the mutant phenotype, while *ZmCCA1a* did not, suggesting that DNA binding activities conferred by the MYB DNA-binding domain are required for the clock function.

### Overexpressing *ZmCCA1b* reduces growth vigor in maize

**Fig 4:**
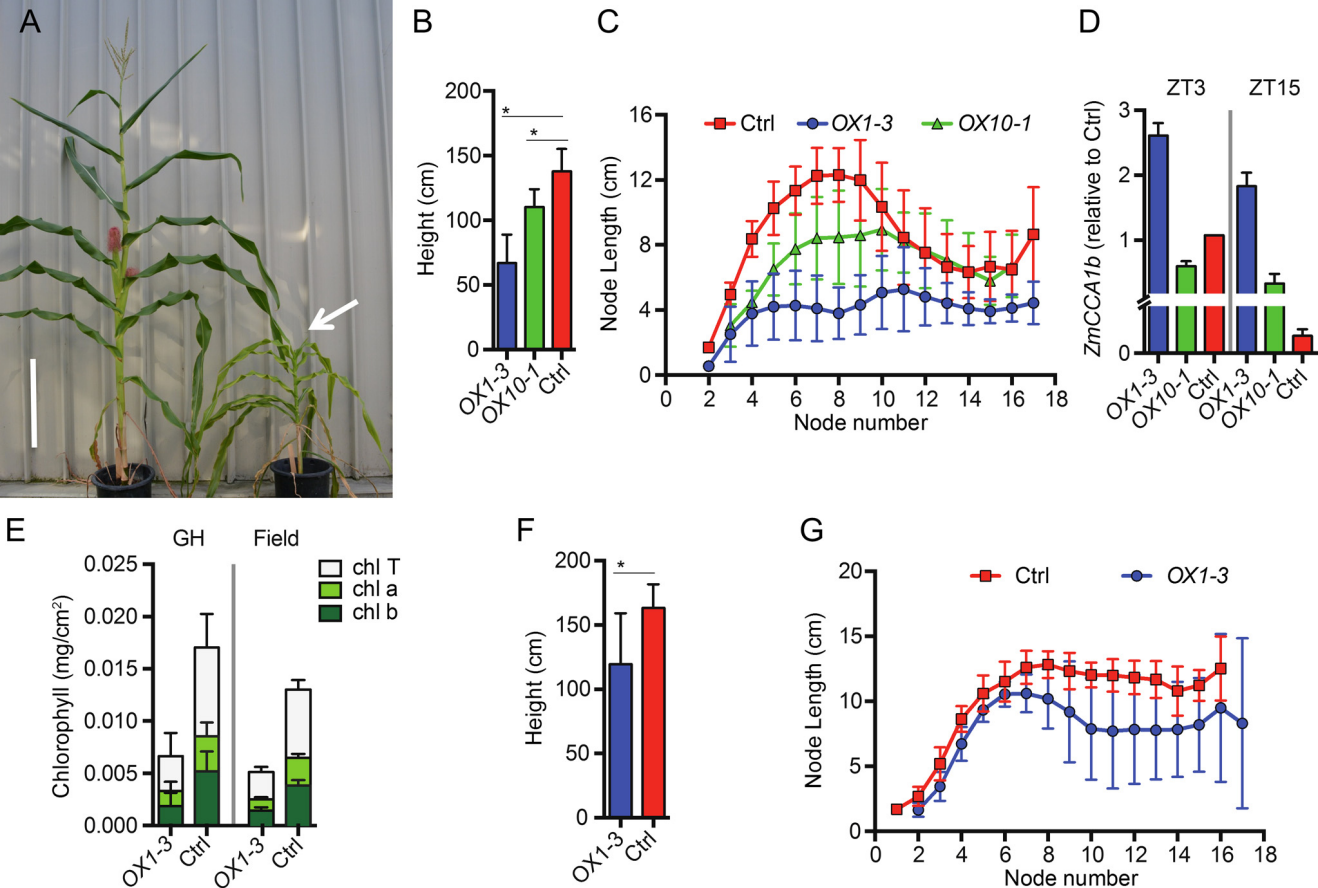
Maize transgenic plants that overexpressed *ZmCCA1b* (*OX1-3* and *OX10-1*). (A) Phenotypes of *OX1-3* and control lines grown in a greenhouse (an arrow indicates the reduced height in *OX1-3*). Scale bar = 40 cm. (B) Height at maturity for *OX1-3*, *OX10-1*, and control plants grown in the greenhouse. (C) Measurement of node elongation for *OX1-3*, *OX10-1*, and control plants grown in the greenhouse (n = 10, ± SD). (D) Relative expression levels (to the control, Ctrl, at ZT3) of *ZmCCA1b* in the leaves of *OX1-3* and *OX1-10* transgenic plants at ZT3 and ZT15 (n = 2, ± SD). Significant difference was calculated using Student’s t-test, *p-value < 0.001. (E) Chlorophyll a (chl a), chlorophyll b (chl b), and total chlorophyll (chl T) content in leaves of *OX1-3* and control plants grown in the greenhouse (GH) or field as indicted. Chlorophyll measurements for greenhouse grown and field-grown plants should not be compared since different developmental stages were tested between the two growth conditions. Greenhouse-grown plants were at stage V9 and field-grown plants were at maturity. (F) Height at maturity for *OX1-3* and control plants grown in the field. Significant difference was calculated using Student’s t-test, *p-value < 0.001. (G) Measurement of node elongation for *OX1-3* and control plants grown in the field (n = 10, ± SD).

To test if *ZmCCA1b* regulates growth vigor in maize, we expressed *ZmCCA1b* driven by the constitutive *UBIQUITIN* promoter-intron cassette (*UBI*) [50] in maize B104 line. Multiple independent transgenic lines were generated; two lines, designated *OX1-3* and *OX10-1*, were analyzed in both greenhouse and field conditions (Fig 4). These lines were maintained as heterozygotes to prevent transgene silencing and were subsequently genotyped to identify the lines carrying the transgene for analysis. In the greenhouse, plant height was dramatically reduced in the *OX* lines (Fig 4A). The *OX1-3* line exhibited a more severe phenotype than the *OX10-1* line (Fig 4B). Reduction in plant height was associated with reduced lengths of early nodes (from #3 to #10), while the total node number was unaffected (Fig 4C). The *OX1-3* line showed more severe reduction in plant height than the *OX10-1* line, which is consistent with higher *ZmCCA1b* expression levels in the former than in the latter (Fig 4D). Moreover, *ZmCCA1* were expressed at higher levels at ZT3 than at ZT15, suggesting oscillation of *ZmCCA1* transcripts in the overexpression transgenic plants. In addition, the amount of total chlorophyll and chlorophyll a and b was reduced in the *OX1-3* line in both greenhouse and field conditions, with more severe reduction in the greenhouse than in the field. In the field, the *OX1-3* line also showed reduced height (Fig 4F), but the reduction in node length occurred in the later nodes (#7 to #16) (compare Fig 4G with 4C). The phenotype was less severe in the field than in the greenhouse. A major difference between the greenhouse and field conditions is the light intensity, suggesting a role of light compensation for altered circadian regulation in growth vigor. Alternatively, enhanced metabolic activities such as increased endogenous sugar levels can provide metabolic feedback to the circadian oscillator, as observed in *Arabidopsis* [51]. These predictions remain to be investigated. Together, these data suggest that disrupting circadian regulation reduces chlorophyll content and growth vigor in maize as in *Arabidopsis* (Fig 3E) [9, 14].

### Maize ZmCCA1s bind to EE and CBS variants and Dof-binding motifs

**Fig 5:**
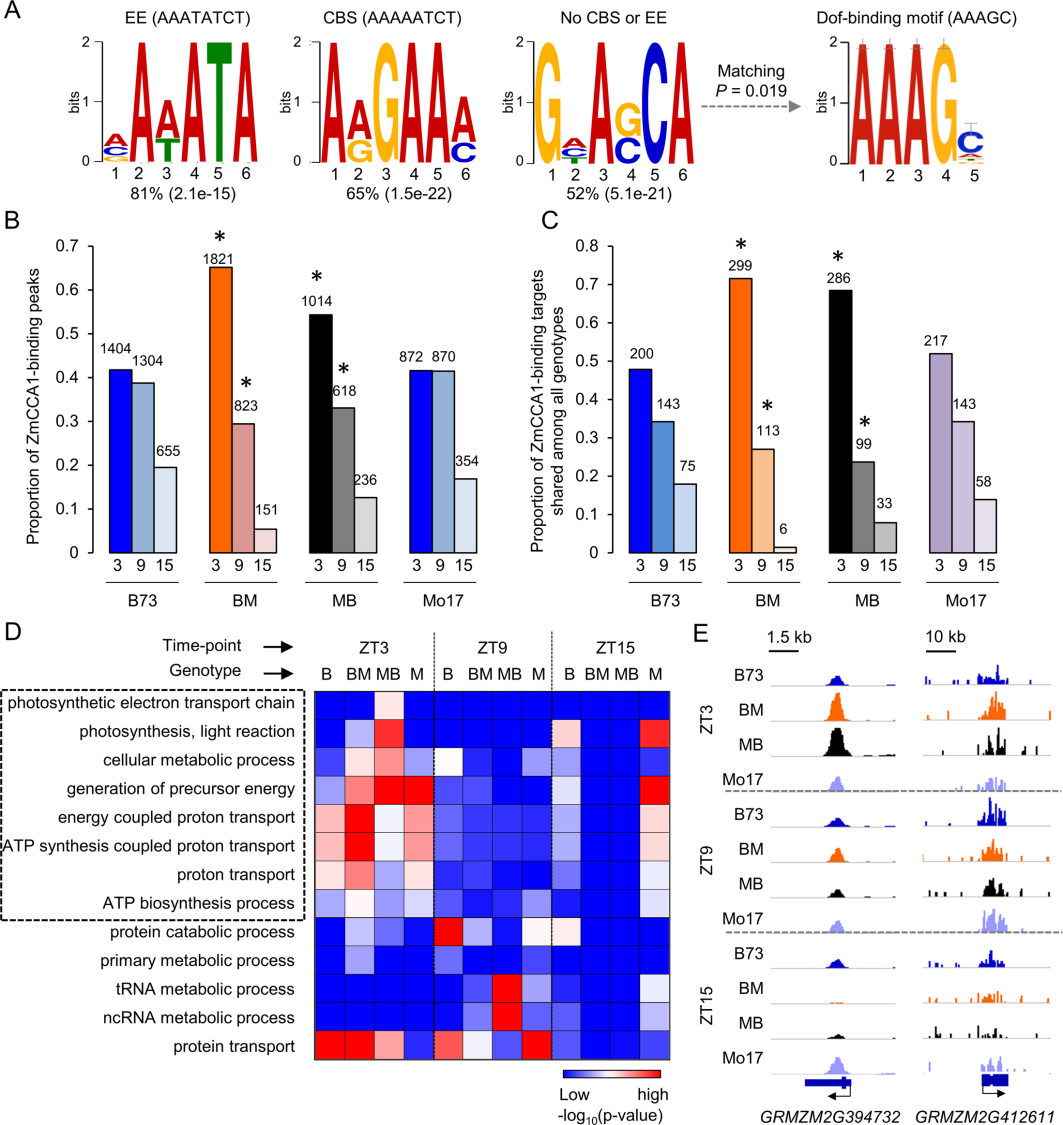
ChIP-seq analysis of ZmCCA1s in the maize inbreds and F1 hybrids. (A) *De novo* motif analysis of ZmCCA1-binding sites using MEME suites showed enrichment of EE, CBS, and no CBS or EE motifs. In the latter, Dof-binding motif was significantly matched in TOMTOM analysis. Percentage (p-value) is shown below each motif. (B and C) Proportion of ZmCCA1-binding peaks (B) and binding targets (C) shared in all genotypes at each time-point. Asterisks indicate statistical significance of ZT3 or ZT9 frequencies between the F1 hybrids and their parents were calculated by Fisher’s exact test. The number of peaks or genes is shown above each bar. (D) Gene Ontology (GO) classification of ZmCCA1-binding targets in each genotype at each time point by GO analysis (false-discovery rate adjusted p-value < 0.05, Hypergeometric test). GO terms associated with carbon fixation are marked by a dashed box. (E) Examples of altered temporal bindings of ZmCCA1s in F1 hybrids compared to the inbred lines. The Y-axis indicates input-subtracted read density on a same-scale for all genotypes and time-points. Arrows indicate gene orientation.

These data indicate that ZmCCA1s, like their homologs in *Arabidopsis*, act as central clock regulators in maize. We predicted that altered *ZmCCA1* expression in the F1 hybrids plays a role in maize heterosis. To test how ZmCCA1s affect expression of output genes and growth vigor in maize, we raised antibodies against the N-terminus MYB DNA-binding domain (S2B Fig), which could recognize ZmCCA1b and a full-length ZmCCA1a if the MYB DNA-binding domain is included. Western blot analysis indicated that accumulation of ZmCCA1s reached high levels in the morning, peaking at ZT3, and decreased during the day (S4A Fig). Using the antibodies, we tested genome-wide ZmCCA1-binding profiles in the F1 hybrids and their parents at ZT3, ZT9 and ZT15 using chromatin immunoprecipitation (ChIP) followed by deep sequencing (ChIP-seq). Sequencing reads were mapped onto the B73 reference genome (AGPv2) and the Mo17 genome, respectively [44] (see Materials and Methods). The paired-end reads that were uniquely and concordantly mapped were normalized among replicates and all genotypes using a “down-sampled” approach [52] and subject to the peak calling pipeline (model-based analysis of ChIP-seq, MACS2, p-value < 0.001) [53] (S4B Fig and S1 Table). The peaks that were present in both replicates (Pearson correlation coefficient = 0.97, based on read coverage over consecutive 1-kb bins) in one or more genotypes were used for further analysis. The number of peaks ranged from 1,874 to 3,364 with a total of 10,136 peaks in all four genotypes (S4C Fig) (S2 Table). Compared to total genomic features, the peaks were enriched in coding, upstream including 2-kb promoter and 5’ untranslated region (UTRs), and downstream flanking sequences (S5A Fig). *De novo* motif analysis identified two top-scoring motifs, namely, AAAATA, an EE (AAATATCT) variant, and AAGAAA, a CBS (AAAAATCT) variant, which represented 81% and 65% of total binding peaks, respectively (Fig 5A). The sequence variants may suggest divergence of canonical EE and CBS sequences between *Arabidopsis* (eudicot) and maize (monocot) over 150–200 million years of evolution [54]. Further analysis using the TOMTOM tool in MEME [55] found a Dof-binding motif (AAAGC), which was statistically significantly similar to the non-classified motif (p-value = 0.019) (Fig 5A). The Dof-transcription factor genes are involved in tissue-specific expression, light signaling, and carbon fixation in maize [42, 56], suggesting that ZmCCA1 interacts with other transcription factors to mediate light-regulated gene expression.

### Temporal regulation of ZmCCA1-binding activities in maize F1 hybrids and inbreds

These peaks were associated with target genes within 10-kb sequences using the filtered gene set (FGS) in MaizeGDB (http://archive.maizegdb.org/cgi-bin/termrefs.cgi?id=2366450). The number of ZmCCA1-binding target genes ranged from 1,406 to 2,511 in each genotype, resulting a non-redundant set of 4,319 target genes (S2 Table), which is consistent with the observation that 10–20% of maize genes exhibit diurnal expression [29, 30], although not all diurnally expressed genes are regulated by ZmCCA1. ZmCCA1s bound to EE and CBS elements of putative circadian clock homologs (S5C Fig), as shown in the EMSA results (Fig 2C). These putative clock genes were nonadditively expressed at certain time points in the hybrids relative to the parents (S6A and S6B Fig). These data suggest a role for ZmCCA1-binding activities in circadian-mediated gene expression. In the reciprocal crosses, more ZmCCA1-binding peaks were found in BM than in MB (S6C Fig), which may suggest a parent-of-origin effect of circadian clock function, consistent with that in *Arabidopsis* intraspecific hybrids [10].

Interestingly, the proportion of ZmCCA1-binding peaks in the hybrids was significantly shifted towards ZT3 (Fisher’s exact test, p-value < 4.3E-15 at ZT3; p-value < 4.2E-05 at ZT9), while the proportion in the inbreds was relatively unchanged between ZT3 and ZT9 (Fig 5B). The ZmCCA1-binding target genes, associated with ZmCCA1-binding peaks, were partitioned into shared among all genotypes and specific to either hybrids or inbreds and among all time-points (S7A Fig) or at each time-point (S7B Fig). Among the shared set of ZmCCA1-binding target genes (418) (significant overlaps; Fisher’s exact test, p-value < 1.59E-179), the proportion was significantly more at ZT3 in the hybrids than in the inbred lines (Fisher’s exact test, p-value < 7.2E-12) but fewer at ZT9 in the hybrids than in the inbred lines (Fisher’s exact test, p-value < 0.03) (Fig 5C). The temporal shift of ZmCCA1-binding target genes in the inbred lines were not statistically significant (Fig 5C), but the shift was significant for the targets that were not shared among all genotypes (S6D Fig). Similarly, among the ZmCCA1-binding target genes that were specific to hybrids or inbreds, the temporal shift was significantly in the hybrids but not in the inbreds (S6E Fig). These data suggest that the ZmCCA1-binding activities of target genes in the hybrids have shifted towards the early morning. However, this does not exclude a possibility of many targets that are preferentially bound by ZmCCA1s in the hybrids compared to the inbreds.

### ZmCCA1-binding genes in Gene Ontology (GO) groups of energy and metabolism

To test the biological function of the temporal shift, we classified ZmCCA1-binding target genes into GO groups (S3 Table). The shared target genes in both hybrids and inbreds were enriched in the genes in electron transport chain, generation of precursor metabolites and energy, photosynthesis, and light reaction among all time-points (S7A Fig) or in different time-points (S7B Fig). This indicates a major role of ZmCCA1s in modulating photosynthetic and metabolic activities, which is consistent with the circadian control of energy and metabolism in other plants [5, 9] and mammals [57]. Moreover, the hybrid-specific target genes were significantly enriched in the genes involved in protein and cellular protein metabolic processes, indicating a role for ZmCCA1s in altered cellular metabolism that would promote growth vigor in maize hybrids. Notably, the inbred-specific target genes among all time points or in different time points were overrepresented in the genes involved in intracellular transport and protein localization (S7 Fig), which could suggest a role for protein stability and movement in maintaining cellular growth and development [4].

Notably, the enrichment of carbon fixation genes in F1 hybrids was almost exclusive at ZT3 (Fig 5D), whereas these genes in the inbreds also occurred in other time-points, in addition to ZT3. The genes in GO terms of other pathways, including protein catabolic process, tRNA metabolic process and protein transport, were also enriched, but they were not associated with the phase-shift (Fig 5D and S3 Table). This suggests a uniform shift in activating photosynthetic and carbon metabolism pathways to the early morning in the hybrids.

### Expression of carbon fixation genes is consistent with the phase-shift of ZmCCA1-binding activities in the hybrids

**Fig 6:**
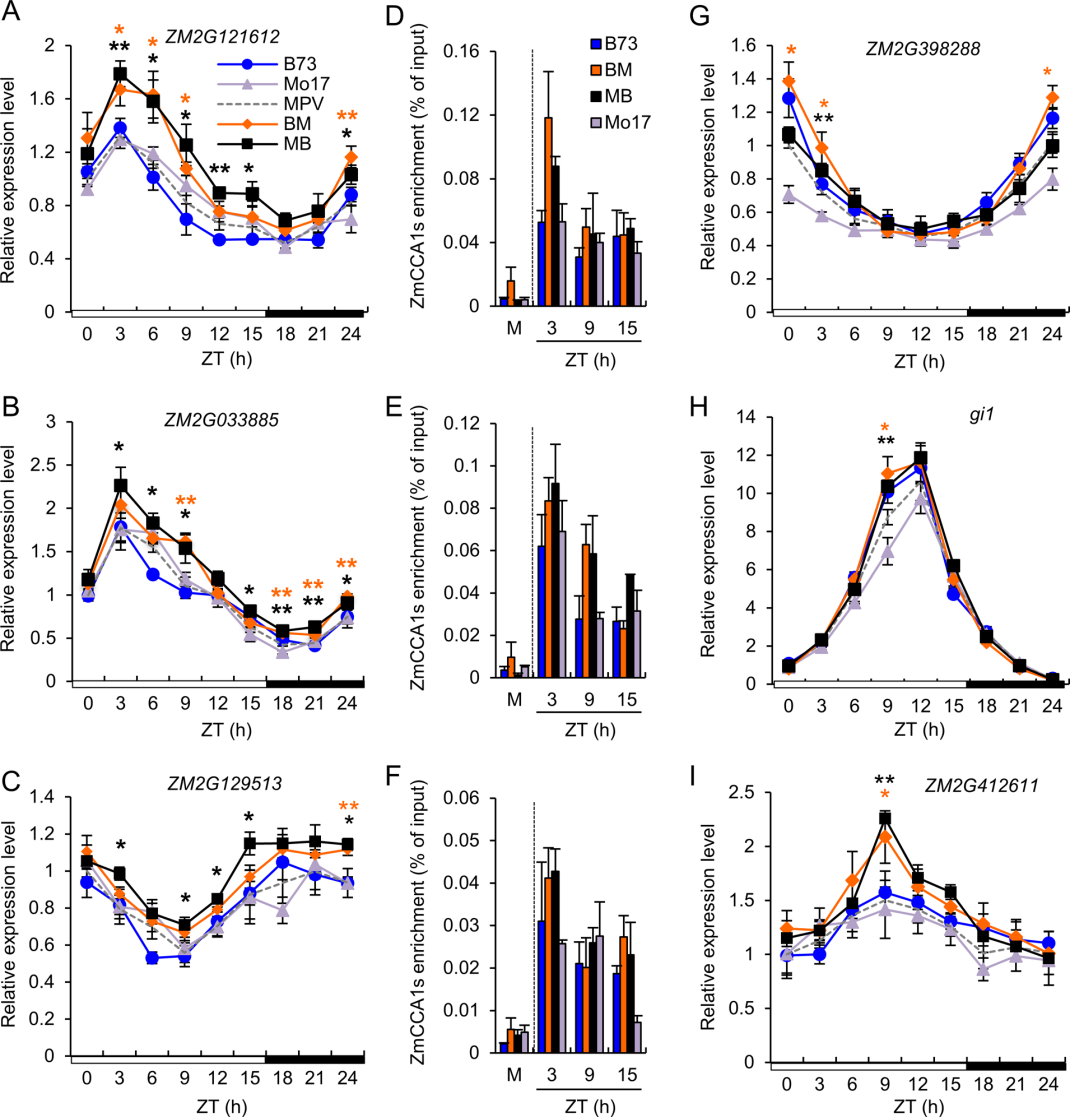
Diurnal expression of ZmCCA1-binding target (carbon fixation) genes in response to the phase-shift of ZmCCA1-binding in the hybrids. (A-C and G) Relative expression levels (means ± SEM, n = 3) of the morning-phased carbon fixation genes, including *ZM2G121612* (A), *ZM2G033885* (B), *ZM2G129513* (C) and *ZM2G398288* (G), every 3 hours in a 24-hour period (light/dark cycle is shown below the histogram). The relative expression level in MPV at ZT0 was set to 1. Significant difference between hybrids and MPV was calculated using Student’s t-test, *p-value < 0.05 and **p-value < 0.01 (D-F) ChIP-qPCR validation of ZmCCA1s ChIP enrichments on *ZM2G121612* (D), *ZM2G033885* (E) and *ZM2G129513* (F). M indicates the enrichment in mock samples at ZT3. The EMSA analysis confirmed ZmCCA1b-bining to the promoter of *ZM2G121612*, *ZM2G033885* and *ZM2G129513 in vitro* (S9 Fig). (H-I) Relative expression levels (means ± SEM, n = 3) of the afternoon-phased genes, *gi1* (H) and *ZM2G412611* (I), every 3 hours in a 24-hour period (light/dark cycle is shown below the histogram). The relative expression level in MPV at ZT0 was set to 1. Significant difference between hybrids and MPV was calculated using Student’s t-test, *p-value < 0.05 and **p-value < 0.01.

Morning phased-genes associated with the temporal shift included those encoding a starch synthase III (*ZM2G121612*), a light harvesting complex photosystem II (*ZM2G033885*), a malate dehydrogenase (*ZM2G129513*), and a starch synthase II (*ZM2G126988*), a putative phospholipid-transporting ATPase (*ZM2G398288*) (Fig 6A–6C and 6G and S8A Fig). These genes were up-regulated in the morning and in the hybrids compared to the inbreds, which correlated with the shift of ZmCCA1-binding activities in the hybrids (Fig 6D–6F and S8E Fig). Thus, the phase-shift of ZmCCA1-binding in the hybrids is positively associated with expression levels of these morning-phased genes. EMSA assays have confirmed the binding activities of ZmCCA1b to the promoters of *ZM2G121612*, *ZM2G033885* and *ZM2G129513* (S9 Fig).

For the afternoon-phased genes, including *gi1* and *ZM2G412611* encoding an alpha-glucan water dikinase chloroplast precursor, their expression peaks were also shifted from ZT12 to ZT9 (Fig 6G–6I), which correlated with the temporal shift of ZmCCA1-binding in the hybrids (S8F Fig). Collectively, these results indicate that altered temporal binding activities of ZmCCA1s to the clock output genes in the maize hybrids are responsible for the expression rhythms of carbon fixation genes, promoting photosynthesis and metabolism.

There were a few exceptions. For example, the expression shift for the genes encoding a photosynthetic reaction center protein (*ZM2G427369*) and a photosystem II reaction center protein Z (*ZM2G394732*) was consistent with the ZmCCA1-binding shift in the hybrids (S8B and S8C Fig), but they were expressed at higher levels in most time points examined. The transcript level of the gene (*ZM2G448142*) encoding an ATP synthase β-subunit in the hybrids was similar to that in the inbreds (S8D Fig). These data suggest that other circadian clock regulators such as TOC1 and ELF3 homologs may also play a role in the diurnal regulation of gene expression in maize hybrids.

### Validation of the temporal shift of ZmCCA1-binding targets in the hybrids

**Fig 7:**
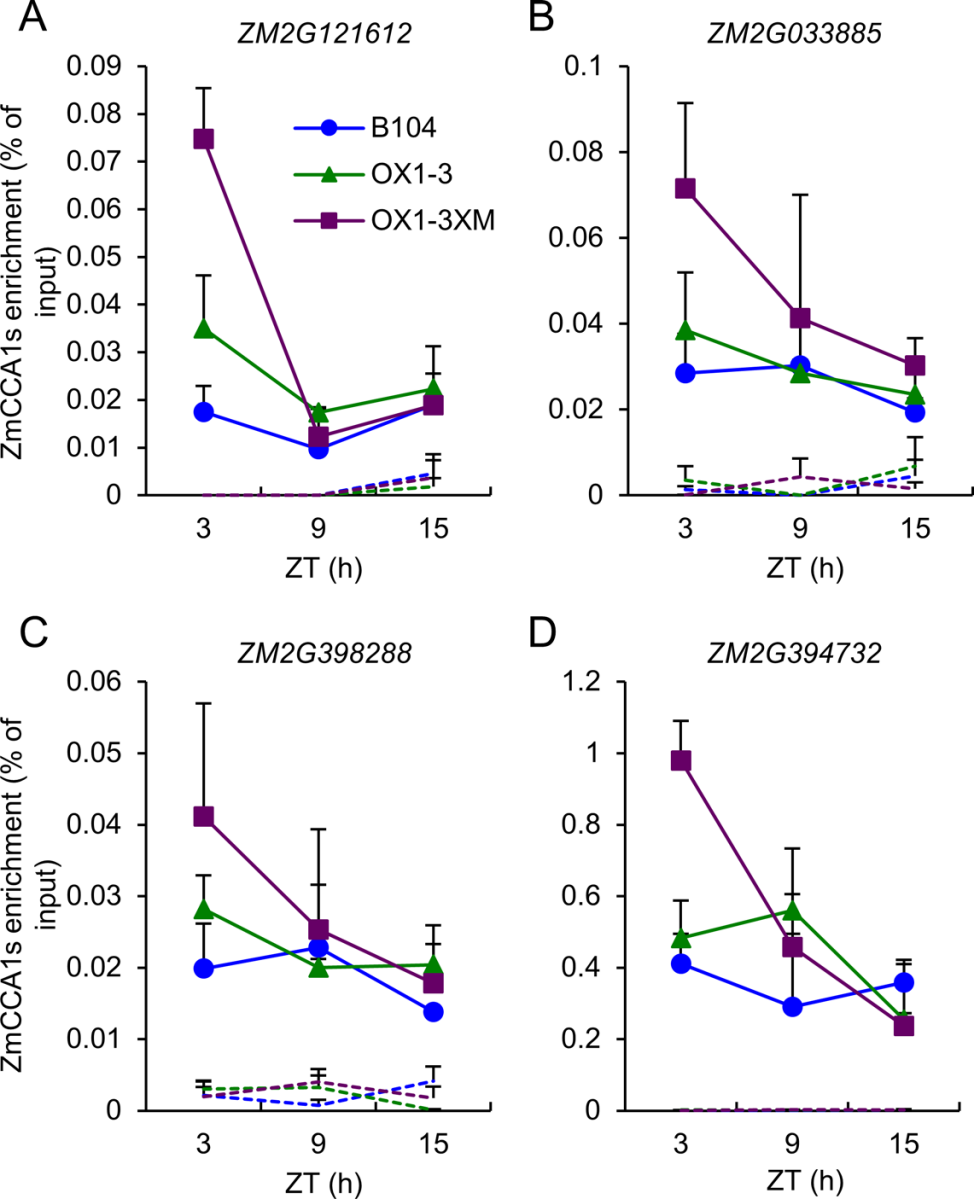
Temporal shift of ZmCCA1-binding to target (carbon fixation) genes in *ZmCCA1b* overexpression line (*OX1-3*) and F1 hybrids. (A-D) ChIP-qPCR assays were performed on ZmCCA1-binding target genes in B104, *OX1-3* and F1 hybrid (*OX1-3*XMo17) lines in four target genes, *ZM2G121612* (A), *ZM2G033885* (B), *ZM2G398288* (C), and *ZM2G394732* (D). ChIP-qPCR values are represented relative to the corresponding input values (means ± SEM, n = 2–3). Dash lines represent ChIP-qPCR data from control (mock) samples.

The ChIP-seq binding activities of ZmCCA1s to the promoters of several carbon fixation genes were confirmed by ChIP-qPCR (Fig 6D–6F) and EMSA (S9 Fig) assays. To test if the temporal shift of ZmCCA1-binding targets is induced in the hybrids, we examined ZmCCA1-binding activities to the promoters of selected four carbon fixation genes in B104, *OX1-3* and *OX1-3*XMo17 by ChIP-qPCR (Fig 7). Compared to the control (B104), overexpressing *ZmCCA1b* in the *OX1-3* increased the binding levels at the four genes tested, consistent with the levels of *ZmCCA1* transcripts that are oscillating in the overexpression lines (Fig 4D). Remarkably, in the F1 hybrid (*OX1-3*XM), the ZmCCA1-binding activities were not only increased in levels but also shifted to the morning-phase (ZT3), which was not obvious in the *OX1-3* line. Although not all possible genotypes were tested, the data suggest a temporal sift of ZmCCA1-binding activities in the hybrids towards early morning, which could play an important role in maize heterosis. Collectively, these results indicate that altered temporal ZmCCA1-binding activities to the clock output genes in the maize hybrids are responsible for expression rhythmicity of carbon fixation genes, promoting photosynthesis, metabolism and growth vigor.

### A developmental role in heterosis

Circadian regulation of gene expression in *Arabidopsis* intraspecific hybrids is established during embryo development and maintained during seedling growth [10], and biomass heterosis is established during early stages of seedling development [58, 59]. Consistent with this notion, expression of the circadian clock genes (*ZmCCA1a* and *ZmCCA1b*) and their output genes in maize hybrids is developmentally regulated during seedling stages (S8G Fig). Expression of *ZmCCA1a*, *ZmCCA1b* and carbon fixation genes was nonadditive in maize hybrids at 5 DAP and 8 DAP but gradually became additive at 11 DAP and 14 DAP. As a control, *Cell Number Regulator 2* (*CNR2*), which controls cell numbers in maize [60], was nonadditively expressed in the hybrids late in seedling development (14 DAP). These data suggest that the influence of circadian regulation on seedling heterosis in maize is likely developmentally regulated, corresponding to gene expression dynamics during maize development [42].

## Discussion

### Developmental regulation and a temporal shift model for heterosis

**Fig 8:**
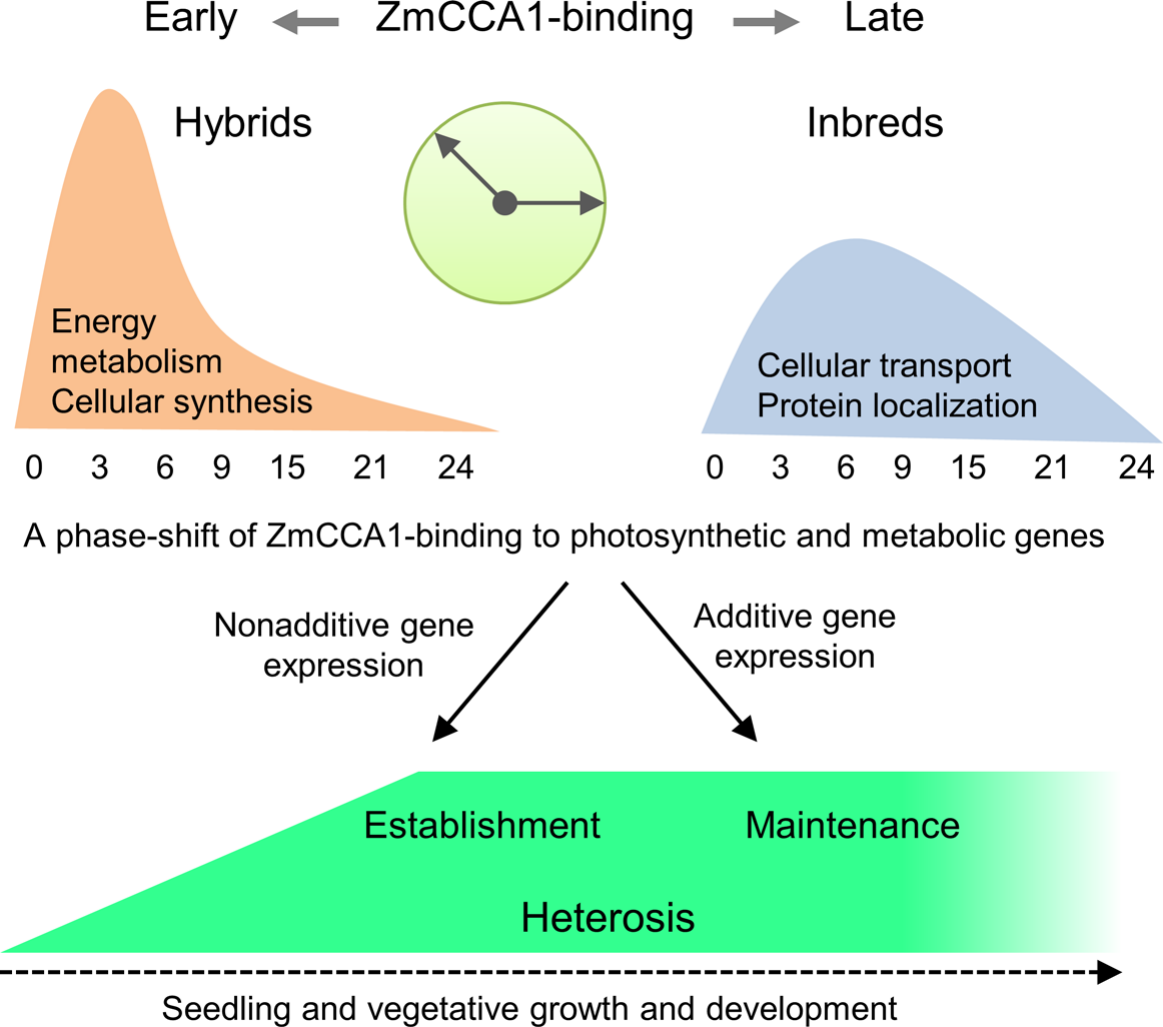
A phase-shift model for heterosis. *ZmCCA1*-regulatory networks orchestrate multiple biological pathways for growth heterosis, which is established at early stage and subsequently maintained during the seedling development. The altered temporal binding of ZmCCA1s to carbon fixation genes in F1 hybrids, which causes nonadditive gene expression, increasing carbon fixation capacity and leading to heterosis. At the stage of maintenance, additive gene expression of the carbon fixation genes is predominant in the hybrids, suggesting a developmental coordination of additive and nonadditive gene expression for growth heterosis. Phase distributions of ZmCCA1-bindings are shown in orange for F1 hybrids and in blue for the inbreds.

Heterosis is predicted to arise from allelic interactions between parental genomes, leading to altered regulatory networks that promote the growth and fitness of hybrids [1, 3, 5]. Here, we demonstrate that one such regulator is the circadian clock in maize hybrids. The circadian clock genes are functionally conserved in *Arabidopsis* and maize. In hybrids, the maize central clock proteins target thousands of output genes early in the morning, including carbon fixation genes. The data collectively support a phase-shift model for heterosis (Fig 8). ZmCCA1-binding target genes are involved in energy and metabolism, which is established early in the seedling and subsequently maintained during growth. During establishment, morning-phased bindings of ZmCCA1s to carbon fixation genes in F1 hybrids (relative to the inbreds) may cause nonadditive gene expression; consequently nonadditive increases in carbon fixation rate, and, ultimately, biomass accumulation. During late stages of development, gene expression in the hybrids is shifted towards additive expression of the circadian-mediated carbon fixation and metabolic genes. This shift of different gene expression modes may explain different findings of additive expression [7], nonadditive, and/or all modes of gene expression [8], which have been documented in maize hybrids or hybrids with different ploidy levels [61]. The temporal shift from non-additive to additive gene expression has also been observed in *A*. *thaliana* F1 hybrids [58, 62]. Moreover, ZmCCA1-binding target genes are dependent on genotypes (inbreds vs. hybrids) (S7 Fig). In the hybrids, coordinated regulation of the protein and cellular metabolic genes could allow them to achieve greater protein metabolic efficiency, as many cellular metabolic processes are simultaneously engaged in production of stable or efficient metabolites, saving energy used in their synthesis and metabolism [4]. Our findings provide novel insights into a circadian-mediated mechanism for the temporal shift of morning-expressed genes in the maize hybrids to promote photosynthetic and metabolic activities, leading to biomass heterosis.

Maize leaf displays a gradient with respect to cell maturation and transcript levels [42, 43]. This developmental gradient is reflected by the differential starch and sugar accumulation between the sink and source within a leaf (Fig 2). In *A*. *thaliana* intraspecific hybrids, intermediate gene expression and higher metabolic activities lead to enhanced performance in the hybrids [58]. This early establishment of heterosis persists over the growth cycle of the hybrids, leading to higher biomass and yield [62]. In maize hybrids, the change from nonadditive gene expression in early developmental stages to additive gene expression in the late stages may serve as a transition or switch to accelerate the rate of development, while the additive gene expression maintains or stabilizes this gradient development at the accelerated rate. The transitional changes in gene expression will enhance metabolic activities that promote higher biomass accumulation and ultimately higher yield in the hybrids.

### Circadian regulation and biomass heterosis in maize

Circadian clocks regulate biological processes of most organisms including plants and animals [57]. Plant growth and development, from stress responses, biomass accumulation, to seedling growth, flowering, and fruit formation, is directly controlled by clock regulators through transcriptional and post-transcriptional regulation of output processes [12, 13]. Physiological activities, including CO_2_ fixation and starch metabolism, are diurnally regulated by circadian clock factors. CCA1 serves as both a transcriptional repressor and activator of target genes, including those in the core circadian oscillator and output pathways [12, 49]. For example, CCA1 is a repressor for evening-phased genes in *A*. *thaliana*; it directly binds to promoters of the evening-phased central clock genes (*TOC1* and *GI*) and represses their expression [63, 64]. In the *cca1-11* mutant, expression of evening-phased starch metabolic genes is up-regulated [9]. Meanwhile, CCA1 is an activator for morning-phased genes; it directly binds to the promoters of the morning-phased central clock genes (*PRR7* and *PRR9*) [65] and cold stress responsive genes [66], which are repressed in *cca1-11* and *cca1 lhy* mutants. CCA1 also regulates expression of morning-phased photosynthetic genes, including *CAB* genes, and their expression is suppressed in the *cca1* mutant [67]. A recent study indicates that circadian-mediated stress-responsive genes could promote and predict heterosis in *Arabidopsis* [68].

Our study has demonstrated functional conservation between *Arabidopsis* and maize CCA1 homologs. Consistent with the positive role of CCA1 in morning-phased genes in *Arabidopsis*, ZmCCA1-bindings to the morning-phased carbon fixation genes were positively correlated with the expression of these genes in the morning (ZT3), whereas ZmCCA1 association with promoters of these genes in the afternoon (ZT9) were negatively correlated with the expression of these genes. It is likely that the positive and negative roles of CCA1 are achieved through its interaction with other factors. For example, in *A*. *thaliana*, CCA1 interacts with LHY *in vitro* and *in vivo*, and the two factors function synergistically in circadian clock regulation [69]. A splice variant of CCA1 (CCA1β), which lacks the MYB DNA-binding domain, interferes with the transcriptional function of CCA1 by interacting with the full-length CCA1 (CCA1α) or LHY to form nonfunctional heterodimers [46]. Without *CCA1β*, the circadian period is longer, whereas overexpressing *CCA1β* leads to a short-period phenotype, suggesting that interacting with spliced CCA1β fine-tunes CCA1α function. In maize, although the long-form of *ZmCCA1a* is not studied, the short-form of *ZmCCA1a* lacking the MYB DNA-binding domain could act as a dominant negative factor to fine-tune circadian rhythms in maize as in *Arabidopsis* [46], as the effect of this splicing variant in *Arabidopsis* overexpressing and complementation tests is relatively small compared to *ZmCCA1b* (Fig 3 and S3 Fig). Both maize *ZmCCA1b* and *ZmCCA1a* are up-regulated in the hybrids in a morning-specific manner with higher expression of *ZmCCA1b*, which may serve as co-regulators for each other with redundant and/or separate functions in the transcriptional regulation of output (photosynthetic, carbon fixation, and metabolic) genes in the hybrids. The rhythmic expression of circadian-mediate genes is likely established over 24-hour diurnal cycles based on *ZmCCA1* expression rhythms (Fig 3).

It is notable that the F1 hybrids share similar molecular and physiological phenotypes such as photosynthetic rate and expression of carbon-fixation genes with one of the parent B73. However, the hybrid still performs better than the best parent, which is known as better-parent heterosis (BPH) [1]. This suggests that some molecular events could be more important than others in promoting growth vigor. For example, net carbon gain (starch and sugar contents) in the sink and source correlates better with the level of heterosis than other molecular and physiological parameters (Fig 2B). Interestingly, the genes corresponding to starch metabolic pathways are regulated by the circadian clock and nonadditively expressed, which could play an important role in heterosis. The circadian-mediated phase-shift model of gene expression in maize hybrids should be further tested using quantification of genome-wide gene expression data, which will demonstrate relative effects of the earlier and extra induction of many morning-phased genes on the contribution to heterosis. In addition, the effect of these morning-phased genes on heterosis could be tested using maize transgenic lines, mutant, and/or recombinant inbred line populations. Further testing the functions of these genes will reveal mechanisms for the circadian clock to regulate unique and complex biological pathways that stimulate growth vigor in maize hybrids.

## Materials and Methods

### Plant materials and growth conditions

The inbred lines B73 and Mo17 and their reciprocal F1 hybrids B73XMo17 (BM) and Mo17XB73 (MB) were used for this study. All maize plants were grown at 600 μmol m^−2^ s^−1^ under 16L:8D (hours of light:dark) cycle with temperature 28°C (light) and 23°C (dark) except for the experiments to measure photosynthetic rates, starch and sucrose content. They were carried out in a glasshouse where natural sunlight (maximum photosynthetically active radiation 598 ± 354 μmol m^−2^ s^−1^, mean ± SD) was supplemented with fluorescent lighting to achieve a 16L:8D cycle with daily maximum temperature at 26.7 ± 2.1°C (mean ± SD), daily minimum temperature at 19.4 ± 0.4°C (mean ± SD), relative humidity at 57 ± 7% (mean ± SD), and net CO_2_ exchange examined during the day. The plants were placed in a randomized design and rotated on a daily basis to minimize positional effects.

### Growth trait measurement

The growth traits included seed mass, aerial biomass, plant height, and leaf-blade length and area. For seed weight, 10 seeds randomly selected from three replicates for each genotype were weighed. For aerial biomass, aboveground seedlings for each genotype were collected at 3, 4, 5 and 6 days after planting (DAP) (n = 4–8). The biomass was weighed after desiccation for 48 h at 80°C. Plant height and leaf-blade length for each genotype were daily measured from 5 DAP to 12 DAP (n = 5). Plant height was measured as the distance from the ground to the top of seedlings. Leaf-blade length was measured as the distance from the base to the tip of the 2^nd^ leaf. The 2^nd^ leaf at 12 DAP for each genotype was photographed and processed for leaf area using NIH ImageJ software (http://imagej.nih.gov/ij/) [70] (n = 6). All experiments were replicated three times, unless noted otherwise.

### *Arabidopsis* plant growth conditions and luciferase assays

*Arabidopsis* seedlings were plated onto Murashige-Skoog media (Sigma, St. Louis, MO) supplemented with 0.8% agar, 3% sucrose (MS agar) and appropriate antibiotics. After stratification in the dark at 4°C for 2 days, plates were transferred to a growth room with a 16L:8D cycle (80 μmol m^−2^ s^−1^) at 22°C. Luminescence recordings were performed on a TopCount (Packard Bioscience, Shelton, CT) over 7 days, as previously described [10]. The data were analyzed by fast Fourier transform–nonlinear least squares (FFT–NLLS) [71] using the interface provided by the Biological Rhythms Analysis Software System version 3.0 (BRASS) (http://www.amillar.org).

### Transgenic plant construction

The construct for *ZmCCA1b* (*ZM2G014902*) overexpression in the binary vector pTF101.1gw1 [72] (generously provided by Dr. Kan Wang, Iowa State University, Ames, IA) consisted the *ZmUBI* promoter-*ZmUBI* intron (*ZmUBI*_*pro*_) cassette immediately upstream of the *ZmCCA1* coding sequence and the *octopine synthase* (*OCS*) terminator immediately downstream. The *ZmUBI*_*pro*_ cassette was PCR amplified from the pANIC6A vector [73] with primers F 5’-CACCGTAAGCTTAATGCAGTGCAGCGTGACCC-3’ and R 5’-GTAAGCTTTGCAGAAGTAACACCAAACAACAGGGTG-3’, which added the HindIII restriction site (underlined) onto each side of the fragment. The *OCS* terminator was PCR amplified from the pANIC6A vector with primers F 5’-CCTGCTTTAATGAGATATGCGAGA CGC-3’ and R 5’-CACCAAAACGACGGCCAGTGCCAA G-3’. The *ZmCCA1b* coding sequence was PCR amplified with primers F 5’-CACCATGGAGGTGAATTCCTCTGGTGAGGAAAC-3’ and R 5’-TTATGTTGATGCTTCACTATCAAGACGAATCCTCTT-3’. The *ZmUBI*_*pro*_ cassette was subcloned into the Hind III site upstream of *attR1* site in pTF101.1gw1. The *ZmCCA1b* coding sequence was moved into with LR Clonase II according to the manufacturer’s recommendations (Thermo-Fisher, Waltham, Massachusetts). The *OCS* terminator was subcloned into the AscI site downstream of the *ZmCCA1b* coding sequence. The Plant Transformation Facility (Iowa State University, Ames, IA) transformed the complete *ZmCCA1b* overexpression binary construct into the B104 maize inbred by Agrobacterium-mediated transformation according to their published protocol [74]. Pollen from T0 plants, which were confirmed to carry at least copy of the one construct, was used for crosses to the B104 inbred. Transgenic lines were maintained in a hemizygous state by crosses to the B104 inbred.

### Plant genotyping

Transgenic plants were screened using fluorescent probe-based endpoint qPCR. Genomic DNA was extracted from leaf tissue taken from the newest expanded leaf when plants were between V3 and V8. The presence of ADH and BAR was assessed together in PCR reactions with primers 5’-TGTTGAGCAGATCTCGGTGAC-3’and 5’-GTTTCTGGCAGCTGGACTTC-3’, with probe 5’-[HEX]AGGACCGGACGGGGCGGTA[BHQ1]-3’, for the *bar* gene in pTF101.1gw1; and primers 5’-GAATGTGTGTTGGGTTTGCAT-3’ and 5’-TCCAGCAATCCTTGCACCTT-3’, with probe 5’-[FAM]TGCAGCCTAACCATGCGCAGGGTA[BHQ1]-3’, for ADH1, which is a single copy gene in the maize genome [75]. Samples with PCR amplification for both ADH and BAR were scored as transgenic and those with ADH alone were scored as non-transgenic.

### Field and greenhouse conditions

Field trials took place at Oxford Tract in Berkeley, CA during summer 2015. The field was sown in a randomized complete block design with two trials separated by one week, planted in late May. In the greenhouse, plants were grown under 16L:8D cycle, supplemented by LumiGrow Pro 325 LED lights (LumiGrow, Inc., Novato, CA), with 25°C days and 20°C nights. Two individual trials were replicated in greenhouse conditions. 5 plants of each family along with 5 non-transgenic siblings were maintained for each trial.

### Plant measurements

In field trials, weekly height measurements were taken beginning at the V8 stage until final height was reached. In greenhouse trials, weekly height measurements were taken starting when plants were at the V7 stage until final height was reached. Height was measured from prop roots to the collar of the last fully expanded leaf. Node length was measured at the end of the field or greenhouse trial after plants had reached maturity. Measurements were taken from the upper prop roots to the final visible node, or the base of the tassel.

### Chlorophyll

Plant tissue for chlorophyll analysis was harvested using a Harris Uni-Core 2.5 mm biopsy punch (Ted Pella, Redding, CA). 4 punches were taken midway from the base to the tip of the leaf, equidistant from the mid-vein and the leaf edge. Tissue was immediately placed in 1 mL DMSO and incubated at 65°C for 30 min. Absorbance at 645 nm and 663 nm was used to determine chlorophyll content based on Arnon’s equation [76].

### Phylogenetic analysis

CCA1 protein sequences are AtCCA1 (At2g46830), AtLHY (At1g01060), SbCCA1 (Sb07g003870), OsCCA1 (Os08g0157600), PnLHY1 (AB429410), PnLHY2 (AB429411), BraA.LHY.a (Bra030496), McCCA1 (AY371287) and GmLCL1 (Glyma11g15580), At: *Arabidopsis thaliana*; Sb: *Sorghum bicolor*; Os: *Oryza sativa*; Pn: *Populus nigra*; BraA: *Brassica rapa*; Mc: *Mesembryanthemum crystallinum*; and Gm: *Glycine max*. The protein sequences were aligned using the ClustalW module [77]. The phylogenetic tree was constructed using the Neighbor-Joining method [78]. The bootstrap values were calculated with 1,000 replicates and shown next to the branches. The evolutionary distances were computed using the Poisson correction method [79] with the units of the number of amino acid substitutions per site. All positions containing gaps and missing data were eliminated. A total of 472 positions were analyzed using MEGA6 [80].

### Gene expression analysis

For gene expression analysis, aerial tissues were harvested and immediately frozen in the liquid nitrogen. Total RNA was extracted from the frozen tissues by PureLink Plant RNA reagent (Invitrogen, Carlsbad, CA) and treated with RQ1 DNaseI (Promega, Madison, WI) according to the manufacturers’ instructions. For cDNA synthesis, 1 μg of DNaseI-treated total RNA was incubated with Omniscript reverse transcriptase (Qiagen, Valencia, CA) in the presence of 10 μM random hexamer (GeneLink, Hawthorne, NY). For qPCR, FastStart Universal SYBR Green Master (ROX) (Roche Applied Science, Indianapolis, IN) was used in the presence of gene-specific primers and template cDNAs in an ABI7500 (Applied Biosystems, Foster City, CA) or LightCycler 96 machine (Roche Applied Science, Indianapolis, IN). The control was *18S rRNA* to estimate the relative expression levels of each gene in three biological replicates. A list of primers for gene expression analysis is provided in S4 Table.

### Photosynthesis, starch and sucrose measurements

Photosynthetic rate was measured on fully expanded 2^nd^ leaves of seedlings at 12 DAP (n = 9), grown under 16L:8D cycle with natural sunlight, every 2 hours from dawn (ZT0) to dusk (ZT14). A LI-6400XT portable photosynthesis analyzer (LICOR Environmental, Lincoln, NE) equipped with a light source (LI-6400-40) and CO_2_ mixer (LI-6400-01) was used to determine net CO_2_ assimilation at a reference CO_2_ concentration of 400 μmol mol^−1^, with cuvette conditions set to match ambient conditions at the start of each measurement period. The experiment was reproduced twice, in February and April 2012, respectively. Data from the April experiment is shown. Starch and sucrose contents were measured on source (middle-tip of the 2^nd^ leaf) and sink (base of the 2^nd^ leaf) of additional replicate plants grown alongside those used for photosynthesis assays. Pooled plants in three biological replicates at ZT14 were used for testing. Leaf discs were collected using an 11 mm-diameter cork borer, weighed and immediately frozen in liquid nitrogen. The frozen tissues were ground, mixed with a homogenization buffer (500 mM MOPS pH 7.5, 5 mM EDTA, 10% ethyl glycol), and then filtered through Miracloth (CalBiochem, San Diego, CA). After centrifugation, pellets were dissolved in DMSO to extract the insoluble carbohydrate fraction, while supernatant was transferred to a new tube as the soluble carbohydrate fraction. The starch content was measured from the insoluble carbohydrate fraction using a commercial assay kit according to the manufacturer’s instruction (R-Biopharm, Darmstadt, Germany). Sucrose content was measured from the soluble carbohydrate fraction using a commercial assay kit according to the manufacturer’s instruction (K-SUFRG Megazyme, Bray, Ireland) and as previously described [9].

### Purification of recombinant ZmCCA1b and ZmCCA1a proteins

The coding sequence (CDS) of *ZM2G474769* (*ZmCCA1a*) was amplified from B73 cDNA by the primer pair 5’-GAATTCATGCCCTTGAGCAATGAG-3’ (*Eco*RІ, underlined) and 5’-GTCGACTCATGTTGATGCTTCACTAT-3’ (*Sal*І). The full-length *ZmCCA1b* (*ZM2G014902*) CDS was amplified from B73 cDNA by the primer pair 5’- GGATCCATGGAGGTGAATTCCTCTGGC-3’ (*Bam*HІ) and 5’- GTCGACTTATGTGGATGCTTCGCTATC-3’ (*Sal*І). The *ZmCCA1a* or *ZmCCA1b* cDNA fragment was cloned into a pGEM-T (Promega, Madison, Wisconsin). After sequence verification, the *ZmCCA1a* or *ZmCCA1b* CDSs was subcloned into pMAL-C2 (New England BioLabs, Beverly, MA) through *Eco*RІ/*Sal*І and *BamH*І/*Sal*І restriction sites, respectively. *E*. *coli* strain Rosetta-gami B competent cells (Novagen, Madison, WI) was used to transform empty pMAL (expressing maltose-binding protein, MBP), pMAL-ZmCCA1a or pMAL-ZmCCA1b, which were grown in 4 ml of Luria-Bertani (LB) media with Carbenicillin (100 mg/L) at 37°C for 18 h. The overnight cultures of Rosetta-gami B cells containing pMAL, pMAL-ZmCCA1a or pMAL-ZmCCA1b construct were diluted into 1:100 in 80 ml LB media with Carbinicillin (100 mg/L) and grown at 37°C to an OD_600_ value of 0.5, when isopropyl-β-D-thiogalactoside (IPTG) (0.1 mM) was added. After 20 h of additional incubation at 16°C, cells were harvested after centrifugation at 4,000 *g* at 4°C for 10 min and resuspended in 2 ml of Column buffer (20 mM Tris-HCl, 200 mM NaCl, 1 mM EDTA). After frozen at -20°C for 18 h, cells were lysed by a Bioruptor Sonicator (Diagenode, Sparta, NJ) and centrifuged at 20,000 *g* at 4°C for 20 min. The cleared cell lysates were diluted 1:5 with Column buffer, and loaded on amylose-coupled agarose resin columns prepared according to the manufacturer’s instruction (New England BioLabs, Beverly, MA). After columns were washed with 12 volumes of Column buffer, MBP, rZmCCA1a and rZmCCA1b were eluted with elution buffer (20 mM Tris-HCl, 200 mM NaCl, 1 mM EDTA, 10 mM Maltose). After filtration by Amicon Ultra 100 K (Millipore, Darmstadt, Germany), the purified MBP, rZmCCA1a or rZmCCA1b was aliquoted and stored at -80°C.

### Chromatin immunoprecipitation (ChIP), ChIP-seq library preparation, and ChIP-PCR

ChIP was performed as previously described with following modifications [81]. Aerial tissues of three biological replicates were harvested at ZT3, ZT9 and ZT15 and were completely submerged in fresh prepared formaldehyde buffer (0.4 M Sucrose, 10 mM Tris-HCl, 1 mM PMSF, 3% Formaldehyde, 5 mM β-mercaptoethanol). A vacuum was applied for 20 min; after adding glycine to a final concentration 125 mM, another vacuum was applied for 5 min. After the formaldehyde/glycine buffer was removed, the cross-linked tissues were washed with sterilized water, briefly dried with paper towels, immediately frozen in liquid nitrogen and stored at -80°C for experimental use. Sonication was performed with 10 cycles of 30 s pulses on and 30 s pulses off to achieve an average fragment size of 400-bp using a Bioruptor Sonicator (Diagenode, Sparta, NJ). Samples were subjected to centrifugation at 13,800 *g* at 4°C for 10 min, and the supernatant containing chromatin was transferred to a new 1.5 ml tube. Immunoprecipitation (IP) was performed using 600 μl of sonicated chromatin with 5 μg of anti-CCA1 antibody. For each IP sample, a mock (no antibody) and input (no IP) were included. Purified DNA (IP, mock and input) from ChIP was resuspended in TE, pH 7.5 and used for qPCR and ChIP-seq library preparation.

ChIP-seq libraries for ChIP and input samples from two biological replicates were constructed using the standard NEB protocol (New England BioLabs, Beverly, MA) using custom made adapters containing barcodes used to pool multiple samples for sequencing. ChIP DNA was subject to end repair, dA-tailing, ligation with the adapters, and amplification by 18 cycles of PCR using Next High-Fidelity 2XPCR Master Mix (New England BioLabs, Beverly, MA). Pair-end (2X100-bp) sequencing was performed on an Illumina HiSeq 2500 at The University of Texas at Austin Genomic Sequencing and Analysis Facility.

For qPCR, purified ChIP and mock DNA was diluted 2 times, and input DNA was diluted 5 times. The diluted DNA (2 μl) was used for qPCR in an ABI7500 machine (Applied Biosystems, Foster City, CA) using FastStart Universal SYBR Green Master (ROX) (Roche Applied Science, Indianapolis, IN). Enrichment of the binding in IP and mock samples was normalized relative to the corresponding input sample. A list of primers for ChIP-qPCR analysis is provided in S4 Table.

### ChIP-seq data analysis

Raw reads were subject to quality trimming and adaptor clipping using FASTX-Toolkit (hannonlab.cshl.edu/fastx_toolkit) followed by removing orphan reads. The filtered pair-end reads were mapped to the maize reference genome (Zmays_284_AGPv2, release 5b.60) and Mo17 genome (reference-guided assembly based on the B73 genome) [44, 82], using Bowtie (version 2.1.0) [83], allowing 1 mismatch in the 20-bp ‘seed’ with options ‘—score-min L,0,-0.3 -X 1000—no-mixed—no-discordant’. Particularly, for B73 and Mo17, the filtered pair-end reads were mapped to the maize B73 reference genome and the Mo17 genome, respectively. For F1 hybrids, the filtered pair-end reads were mapped to both B73 and Mo17 reference genomes, and the best alignment was selected for each read. Only reads concordantly mapping to the genome exactly 1 time was kept and used for peak calling. Duplicate reads were removed from the Bowtie output using samtools rmdup command [84]. To normalize sequencing depth of the mapped reads among samples from different genotypes and time-points, we adopted a normalization method based on the previously published paper [52]. In order to adjust different sequencing depths among genotype and time-points, the uniquely and concordantly mapped paired-end reads were “down-sampled” to the lowest number of samples without PCR duplicates (S1 Table). For example, if B73, Mo17, BM and MB had 4, 5, 6 and 7 million reads respectively, all samples were down-sampled to 4 million reads. This will ensure that numbers of binding peaks are comparable among genotypes and time-points. The criteria for identifying targets were based on abundance of peak enrichment using ANOVA test (p-value < 0.05 as a cut-off). When target genes showed same abundance 2 or more time-points, they were excluded from the phase-shift analysis.

The latest version of model-based analysis of ChIP-seq algorithm (MACS, version 2.0.1) [53] was used to identify enriched peaks on each ChIP-seq file against the corresponding input file using a mappable genome size of–g 2.07e+09 and cut off p-value of 1e-3. Peaks were defined if they overlapped in two biological replicates. To make a master-peak list from the three time-points, the peaks obtained from each time-point were merged for each genotype. Integrated Genomics Viewer (version 2.3.46) [85] was used to visualize duplicate-filtered input subtracted ChIP signals. For the normalization, modules of the deepTools suite (http://deeptools.ie-freiburg.mpg.de) [86] were used. *De novo* motif analysis was performed with the total master-peaks using MEME suite [87] in which JASPAR CORE (2014) plants database was selected for motif database. GO enrichment analysis was performed using the web interface of agriGO (http://bioinfo.cau.edu.cn/agriGO/) [88] with false-discovery rate adjusted p-value < 0.05 (Hypergeometric test) as a cutoff. Biological process among ontology categories was used. The heatmap for GO analysis was generated using R package gplots. Peak distribution was determined with respect to the maize gene model (release 5b.60).

### Generation of *Arabidopsis* transgenic lines

To create *CAB2*:*LUC*, a native *Arabidopsis CAB2* (*AT1G29920*) promoter fragment was amplified from Col-0 wild-type genomic DNA by the primer pair 5’- CTCGAGTTATATTAATGTTTCGATCATC-3’ (*Xho*І) and 5’- CCATGGGTTCGATAGTGTTGGATTATA-3’ (*Nco*І) and cloned into pGEM-T (Promega, Madison, WI). After sequence validation, the *CAB2* promoter fragment was subcloned into pFAMIR binary vector (Basta resistance), which was fused with *LUC* CDS, via *Xho*І/*Nco*І restriction sites. The *CAB2*:*LUC* construct was transformed into the Col-0 and WS wild-types, respectively, using a floral dip method [89]. Homozygous T3 lines in each background were selected for the following analysis. To generate *CCA1-OX*, *ZmCCA1-OX* and *ZmCCA1b-OX* transgenic lines, *CCA1*, *ZmCCA1a* and *ZmCCA1b* cDNAs were amplified by the following primer pairs, respectively, *CCA1* 5’-CTCGAGATGGAGACAAATTCGTCTGG-3’ (*Xho*І) and 5’- GGATCCTCATGTGGAAGCTTGAGTTTC-3’ (*Bam*HІ), *ZmCCA1a* 5’- CTCGAGATGCCCTTGAGCAATGAG-3’ (*Xho*І) and 5’- GGATCCTCATGTTGATGCTTCACTATC-3’ (*Bam*HІ), and *ZmCCA1b* 5’- CAAGCTCGAGATGGAGGTGAATTCCTCTGGC-3’ (*Xho*І) and 5’- GGATCCTTATGTGGATGCTTCGCTATC-3’ (*Bam*HІ), and cloned into pGEM-T (Promega, Madison, WI). After sequence validation, *CCA1* CDS, *ZmCCA1a* CDS or *ZmCCA1b* CDS was subcloned into pF35SE binary vector (conferring Kanamycin resistance) through *Xho*І/*Bam*HІ restriction sites. The resulting constructs designated *CCA1-OX*, *ZmCCA1a-OX* and *ZmCCA1b-OX*, respectively. The construct of vector control, *CCA1-OX*, *ZmCCA1-OX* or *ZmCCA1b-OX* was introduced into the transgenic Col-0 plants that express *CAB2*:*LUC*. T2 lines were used for the luciferase and biomass analysis. For the *cca1-11* complementation analysis with maize *CCA1* homologs, a native *Arabidopsis CCA1* (*AT2G46830*.*1*) promoter fragment was amplified from WS wild-type genomic DNA by the primer pair, 5’- GAATTCGCCACGTCCTTCCTTCAATC-3’ (*Eco*RІ) and 5’- CTCGAGCACTAAGCTCCTCTACACAA-3’ (*Xho*І), and cloned into pGEM-T (Promega, Madison, WI). After sequence validation, the *CCA1* promoter fragment was subcloned into pFAMIR binary vector (Hygromycin resistance), via *Eco*RІ/*Xho*І restriction sites. To create *CCA1*:*CCA1*, the *CCA1* CDS was amplified from the WS cDNA by the primer pair, 5’- ATTTAAATATGGAGACAAATTCGTCTGG-3’ (*Swa*І) and 5’- GGATCCTCATGTGGAAGCTTGAGTTTC-3’ (*Bam*HІ), and cloned into pGEM-T (Promega, Madison, WI). To generate *CCA1*:*ZmCCA1a*, the *ZmCCA1a* CDS was amplified from *Z*. *mays* B73 cDNA by the primer pair, 5’- CTCGAGATGCCCTTGAGCAATGAG-3’ (*Xho*І) and 5’- GGATCCTCATGTTGATGCTTCACTATC-3’ (*Bam*HІ), and cloned into the pGEM-T (Promega, Madison, WI). To generate *CCA1*:*ZmCCA1b*, the *ZmCCA1b* CDS was amplified from *Z*. *mays* B73 cDNA by the primer pair, 5’- CAAGCTCGAGATGGAGGTGAATTCCTCTGGC-3’ (*Xho*І) and 5- ACCGGATCCTTATGTGGATGCTTCGCTATC-3’ (*Bam*HІ), and cloned into the pGEM-T (Promega, Madison, WI). After sequence validation, *CCA1* CDS, *ZmCCA1a* CDS or *ZmCCA1b* CDS was subcloned into the pFAMIR (Hygromycin resistance), which harbors the *CCA1* promoter fragment, through the respective restriction sites, generating *CCA1*:*CCA1*, *CCA1*:*ZmCCA1a*, and *CCA1*:*ZmCCA1b* constructs, respectively. The construct of *CCA1*:*CCA1*, *CCA1*:*ZmCCA1a* or *CCA1*:*ZmCCA1b* was transformed into *A*. *thaliana cca1-11* plants (obtained from the Arabidopsis Biological Resources Center, ABRC, CS9865), which express *CAB2*:*LUC*, generating *CCA1*:*CCA1 cca1-11*, *CCA1*:*ZmCCA1a cca1-11* or *CCA1*:*ZmCCA1b cca1-11* transgenic lines, respectively. T1 transgenic lines were selected for the luciferase analysis.

### Electrophoretic mobility shift assays (EMSA)

DNA probes were generated by annealing PAGE-purified sense and antisense oligonucleotides (S5 Table). The double-stranded oligonucleotides were [^32^P] end-labeled using a T4 Polynucleotide Kinase according to the manufacturer’s instruction (New England BioLabs, Beverly, MA). The recombinant proteins (0.5 to 2 pmol) were mixed with 20 fmol of the radiolabeled probes, without or with variable amounts of unlabeled competitor DNA in reaction buffer (25 mM HEPES-KOH pH7.5, 2.5 mM DTT, 75 mM KCl, 10% glycerol, 1.25 ng poly-dIdC). Each reaction was incubated on room temperature for 10 min without the probes and then incubated on ice for 20 min with the radiolabeled probes. The competitor concentrations were at 0 (-), 0.5 (25X), 1 (50X) and 2 (100X) pmol. After the incubation, the reaction mixtures were resolved by electrophoresis on a 5% non-denaturing polyacrylamide gel. Gels were dried in a gel dryer (Bio-Rad, Richmond, CA) and exposed to X-ray film (Kodak, Rochester, NY).

### Western blot analysis

Total crude protein was extracted from the aerial tissues of seedlings at 5 DAP using an extraction buffer (50 mM Sodium Phosphate pH 8.0, 1 mM EDTA, 5 mM DTT, 10% Glycerol). 30 μg of total crude proteins were resolved in 10% SDS-PAGE gel and transferred to PVDF membrane (GE Healthcare, Piscataway, NJ). ZmCCA1s were detected using anti-CCA1 antibody (1:2,000) that was generated against the N-terminus MYB DNA-binding domain (epitope: residues 11–77 of ZmCCA1s, S2B Fig), followed by HRP-conjugated anti-rabbit secondary antibody (1:20,000) (12–348 Upstate, Lake Placid, NY). Loading control was detected using an anti-H3 antibody (1:5,000) (Ab1791 Abcam, Cambridge, MA). Immunoreactive protein was visualized on X-ray film (Kodak, Rochester, NY) using SuperSignal West Pico Substrate (Thermo Scientific, Waltham, MA)

## Supporting Information

S1 Fig The early-established heterosis is subsequently maintained. (A) The percent better-parent heterosis (BPH) is shown for plant height. The percentage BPH (means ± SEM) was calculated for each biological replicate (n = 5) as: %BPH = [(Hybrid–Best parent)/Best parent] X 100. (B) Representative growth vigor in the reciprocal hybrids at different developmental stages; 5 DAP, 8 DAP, 11 DAP and 14 DAP. Scale bar, 40 mm.
(PDF)

S2 Fig Phylogenetic tree and multi-alignment of CCA1 homologs in plants, and gene expression patterns in maize tissues. (A) Phylogenetic tree of CCA1/LHY homologs in plants. The Neighbor-Joining phylogenetic tree of CCA1/LHY was constructed from amino acid sequences, and bootstrap values calculated with 1,000 replicates are shown next to the branches. (B) Multiple sequence alignment showing N-terminus MYB DNA-binding domain of CCA1 homologs in plants. Amino acid sequences were aligned using the ClustalW module. The MYB-DNA binding domain, indicated by the black bar, is highly conserved in the CCA1 homologs. The red bar indicates the region recognized by anti-CCA1 antibody. Consensus match is plotted below. Representatives are shown from monocots (Os, O. sativa; Sb, S. bicolor; Zm, Z. mays) and eudicots (At, A. thaliana; Bra, B. rapa; Mc, M. crystallinum; Pn, P. nigra; Gm, Glycine max). (C) Expression levels of the two maize *CCA1* paralogous genes in 17 tissues of B73. The maize gene atlas developed by RNA-seq was used to compare the tissue-specific expression for *ZmCCA1a* and *ZmCCA1b*. Expression level indicates fragments per kilobase pair of exon model per million fragments mapped (FPKM).
(PDF)

S3 Fig Circadian characterization of maize CCA1 homologs in *Arabidopsis*. (A) A plot of period versus relative amplitude error (RAE) of *CAB2*:*LUC* activity in wild-type (Col-0), *Vec-1*, *ZmCCA1a-OX7*, *ZmCCA1b-OX2* and *CCA1-OX2* under LL (means ± SEM). RAE > 0.6 represents arrthymicity. Mean values were compared to those of the vector control line using Student’s t-test. Single and double asterisks indicate significant differences at p-value < 0.05 and p-value < 0.01, respectively. T2 plants were used in the analysis. (B) *CAB2*:*LUC* activity rhythms in wild-type (WS), *cca1-11*, *CCA1*:*ZmCCA1a cca1-11*, *CCA1*:*ZmCCA1b cca1-11* and *CCA1*:*CCA1 cca1-11* under LL (means ± SEM, n = 4–8). White and grey bars represent the subjective day and night, respectively. T1 plants were used in the analysis. T1 plants were used in the analysis. (C) A plot of period versus relative amplitude error (RAE) of *CAB2*:*LUC* activity in wild-type (WS), *cca1-11*, *CCA1*:*ZmCCA1a cca1-11*, *CCA1*:*ZmCCA1b cca1-11* and *CCA1*:*CCA1 cca1-11* under LL (means ± SEM). Mean values were compared to those of *cca1-11* using Student’s t-test, *p-value < 0.05 and **p-value < 0.01.
(PDF)

S4 Fig Specificity of antibody against ZmCCA1s and computational pipeline for ChIP-seq analysis. (A) Immunodetection of ZmCCA1s in the maize inbreds and hybrids using the polyclonal antibodies used for our ChIP-seq. Histone H3 protein was used as a loading control. Plant tissues were collected at time points as labeled under the diurnal condition and used for protein extraction. (B) Workflow of computational analysis of ZmCCA1s ChIP-seq. Summary of mapping statistics is provided in S1 Table. (C) Line graph displays pairwise correlations between biological replicates calculated by Pearson correlation coefficients in windows of 1-kb over mapped read files.
(PDF)

S5 Fig Relative distribution of ZmCCA1-binding peaks across genomic regions and binding motifs found in the peaks. (A) Histogram showing genomic annotation of ZmCCA1-binding peaks. Genome indicates the maize genome fraction as a control. Significant differences between Genome and each genotype are shown above each category using Fisher’s exact test. (B) Local enrichment of ZmCCA1-binding at putative maize clock genes at ZT3, ZT9 and ZT15. The Y-axis indicates input-subtracted read density on a same-scale for all genotypes and time-points. Arrows indicate gene orientation.
(PDF)

S6 Fig Temporal regulation of ZmCCA1-binding targets and nonadditive expression of putative clock homolog genes. (A-B) Relative expression levels (means ± SEM, n = 3) of *ZmTOC1a* (A) and *ZmPRR59* (B) every 3 hours in a 24 h period analyzed by qRT-PCR (light/dark cycle is shown below the histogram). Significant difference between hybrids and MPV was calculated using Student’s t-test, *p-value < 0.05 and **p-value < 0.01. The relative expression level in MPV at ZT0 was set to 1. (C) Hierarchically clustered heatmap showing the ChIP signals of ZmCCA1-binding (Z-scores) on ZmCCA1-binding peaks of each genotype. (D) Proportion of ZmCCA1-binding targets that were not shared. Statistical differences of ZT3 or ZT9 frequencies between the inbreds and F1 hybrids were calculated using Fisher’s exact test. Blue and purple values indicate the comparison with B73 and Mo17, respectively. The number of peaks or genes is shown above each bar. (E) Proportion of ZmCCA1-binding targets that were specific to either hybrids or inbreds. Statistical differences of ZT3, ZT9 or ZT15 frequencies between the hybrid-specific and inbred-specific target genes were calculated using Fisher’s exact test (ZT3, p-value = 2.2E-15; ZT9, p-value = 3.6E-15; ZT15, p-value = 2.9E-15). The number of genes is shown above each bar.
(PDF)

S7 Fig Diverse biological pathways are enriched in ZmCCA1-binding target genes. (A) Venn diagram showing genotype-dependent and -shared ZmCCA1s targets in the inbreds and hybrids (B73, n = 2521; Mo17, n = 1522; BM, n = 2063; MB, n = 1406). (B-D) Venn diagram showing genotype-dependent and -shared ZmCCA1s targets at ZT3 (B), ZT9 (C) or ZT15 (D). Inbred-specific or hybrid-specific genes are indicated by dashed circles. Enriched functional pathways in GO analysis are shown with false-discovery rate adjusted p-value (Hypergeometric test). Full lists of GO terms are available in S3 Table.
(PDF)

S8 Fig Temporal shift of ZmCCA1-binding target (carbon fixation) genes and their expression. (A-D) Relative expression levels (means ± SEM, n = 3) of *ZM2G126988* (A), *ZM2G427369* (B), *ZM2G294732* (C), and *ZM2G448142* (D) every 3 hours in a 24-hour period (light/dark cycle is shown below the histogram). The relative expression level in MPV at ZT0 was set to 1. Significant difference between MPV and hybrids was calculated using Student’s t-test, *p-value < 0.05 and **p-value < 0.01. The right panel for each gene shows ZmCCA1-binding peaks at ZT3, ZT9 and ZT15. The Y-axis indicates input-subtracted read density on a same-scale for all genotypes and time-points. Arrows indicate gene orientation. (E-F) ZmCCA1-binding enrichments of *ZM2G398288* (E) and *gi1* (F) at ZT3, ZT9 and ZT15. Notations are the same as above. (G) Heatmap of qRT-PCR data (|log_2_(F1/MPV)|, n = 3) showing nonadditive expression of maize *CCA1* and carbon fixation genes in the hybrids at ZT3 except for *ZM2G412611* at ZT9. *CNR2* was used as a marker for nonadditive expression in the seedlings.
(PDF)

S9 Fig Verification of ZmCCA1-binding to promoters of carbon fixation genes *in vitro*. Binding of recombinant ZmCCA1b (rZmCCA1b) to the promoters of ZmCCA1-binding target genes associated with carbon fixation *in vitro*. Radioisotope-labeled DNA probes (endogenous promoter fragments) were incubated in the presence of MBP (1 pmol), rZmCCA1a (1 pmol), and rZmCCA1b (0.5, 1 and 2 pmol). Shifted protein-DNA complexes are indicated by the arrow. Competitors: 25X, 50X and 100X molar excess of unlabeled prompter DNA. M: DNA in which EE or CBS site was mutated; N: no EE or CBS site in the DNA fragment. Location of probes for each gene is shown below the gel image. Arrowheads represent EE or CBS site. Numbers are relative to the transcription start site (+1) and 5’ UTR (grey box).
(PDF)

S1 Table ChIP-seq mapping summary. (DOCX)

S2 Table Genomic loci and annotated genes associated with ZmCCA1-binding peaks. (XLSX)

S3 Table Enrichment of Gene Ontology (GO) groups of ZmCCA1-binding targets. (XLSX)

S4 Table List of primers used in qRT-PCR and ChIP-qPCR. (DOCX)

S5 Table List of oligonucleotides used in EMSA. (DOCX)
