# Supplementary material for: Temporal Shift of Circadian-Mediated Gene Expression and Carbon Fixation Contributes to Biomass Heterosis in Maize Hybrids
Source: PLoS Genet. 2016 Jul 28;12(7):e1006197. doi: 10.1371/journal.pgen.1006197 (PMC4965137; doi:10.1371/journal.pgen.1006197)
Supplement: S1 Fig — (A) The percent better-parent heterosis (BPH) is shown for plant height. The percentage BPH (means ± SEM) was calculated for each biological replicate (n = 5) as: %BPH = [(Hybrid–Best parent)/Best parent] X 100. (B) Representative growth vigor in the reciprocal hybrids at different developmental stages; 5 DAP, 8 DAP, 11 DAP and 14 DAP. Scale bar, 40 mm. (PDF) [file pgen.1006197.s001.pdf]

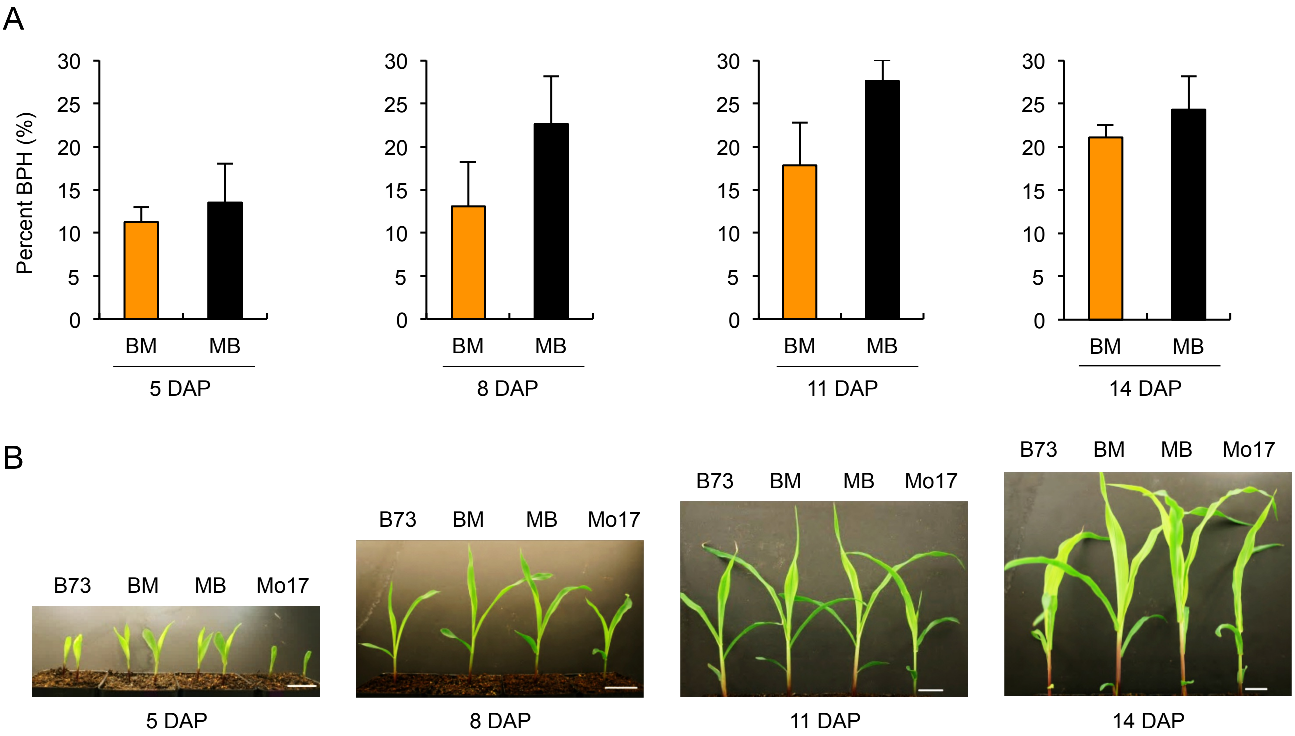

S1 Fig. The early-established heterosis is subsequently maintained. (A) The percent better-parent heterosis (BPH) is shown for plant height. The percentage BPH (means ± SEM) was calculated for each biological replicate (n = 5) as: %BPH = [(hybrid – better-parent)/better-parent] X 100. (B) Representative growth vigor in the reciprocal hybrids at different developmental stages; 5 DAP, 8 DAP, 11 DAP and 14 DAP. Scale bar, 40 mm.
