# Supplementary material for: Temporal Shift of Circadian-Mediated Gene Expression and Carbon Fixation Contributes to Biomass Heterosis in Maize Hybrids
Source: PLoS Genet. 2016 Jul 28;12(7):e1006197. doi: 10.1371/journal.pgen.1006197 (PMC4965137; doi:10.1371/journal.pgen.1006197)
Supplement: S2 Fig — (A) Phylogenetic tree of CCA1/LHY homologs in plants. The Neighbor-Joining phylogenetic tree of CCA1/LHY was constructed from amino acid sequences, and bootstrap values calculated with 1,000 replicates are shown next to the branches. (B) Multiple sequence alignment showing N-terminus MYB DNA-binding domain of CCA1 homologs in plants. Amino acid sequences were aligned using the ClustalW module. The MYB-DNA binding domain, indicated by the black bar, is highly conserved in the CCA1 homologs. The red bar indicates the region recognized by anti-CCA1 antibody. Consensus match is plotted below. Representatives are shown from monocots (Os, O. sativa; Sb, S. bicolor; Zm, Z. mays) and eudicots (At, A. thaliana; Bra, B. rapa; Mc, M. crystallinum; Pn, P. nigra; Gm, Glycine max). (C) Expression levels of the two maize CCA1 paralogous genes in 17 tissues of B73. The maize gene atlas developed by RNA-seq was used to compare the tissue-specific expression for ZmCCA1a and ZmCCA1b. Expression level indicates fragments per kilobase pair of exon model per million fragments mapped (FPKM). (PDF) [file pgen.1006197.s002.pdf]

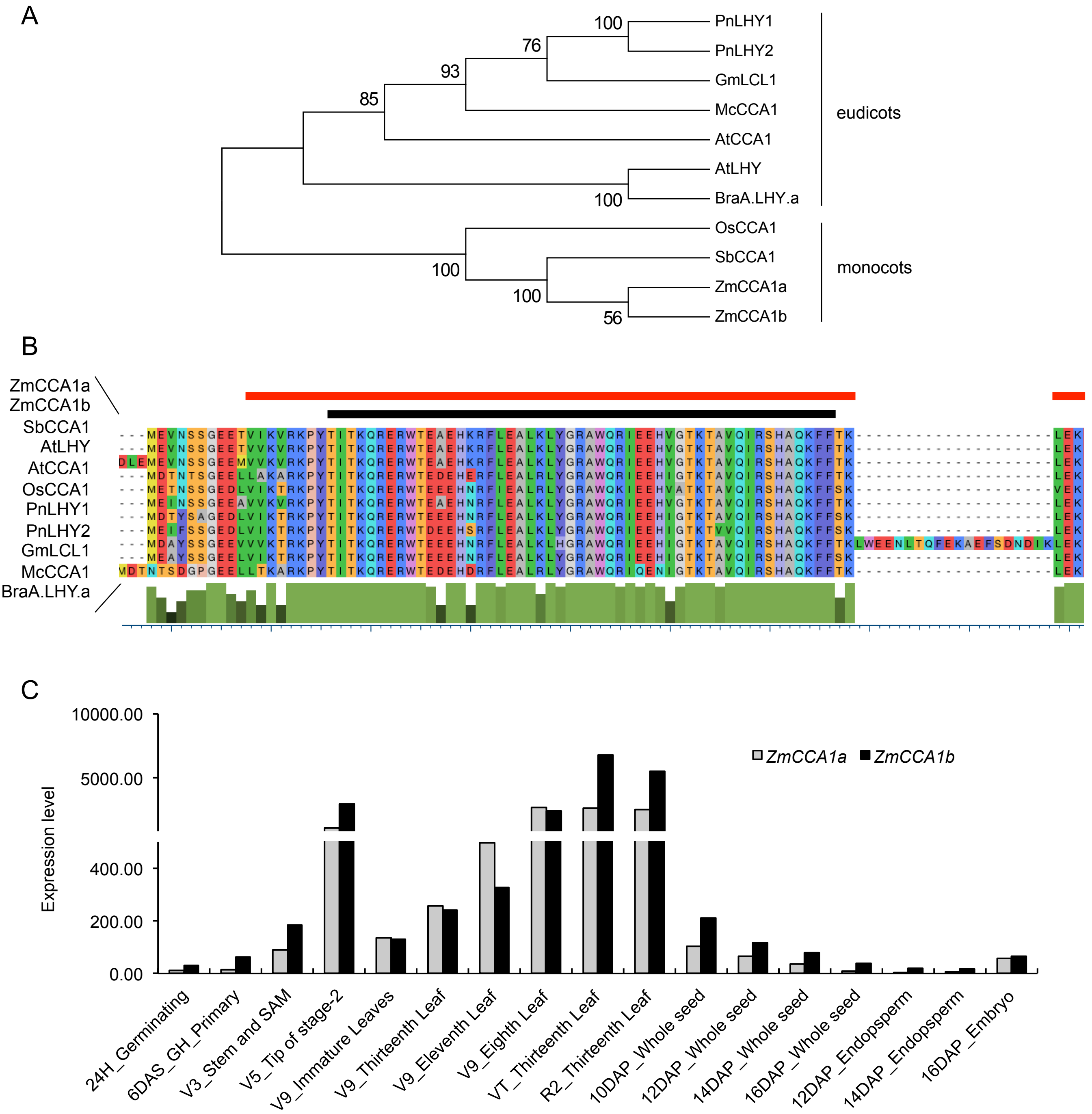

S2 Fig. Phylogenetic tree and multi-alignment of CCA1 homologs in plants, and gene expression patterns of *ZmCCA1a* and *ZmCCA1b* in maize tissues. (A) Phylogenetic tree of CCA1/LHY homologs in plants. The Neighbor-Joining phylogenetic tree of CCA1/LHY was constructed from amino acid sequences, and bootstrap values calculated with 1,000 replicates are shown next to the branches. (B) Multiple sequence alignment showing N-terminus MYB-DNA binding domain of CCA1 homologs in plants. Amino acid sequences were aligned using the ClustalW module. The MYB-DNA binding domain, indicated by the black bar, is highly conserved in the CCA1 homologs. The red bar indicates the region recognized by anti-CCA1 antibody. Consensus match is plotted below. Representatives are shown from monocots (Os, O. sativa; Sb, S. bicolor; Zm, Z. mays) and eudicots (At, A. thaliana; Bra, B. rapa; Mc, M. crystallinum; Pn, P. nigra; Gm, Glycine max). (C) Expression levels of the two maize *CCA1* paralogous genes in 17 tissues of B73. The maize gene atlas developed by RNA-seq was used to compare the tissue-specific expression for *ZmCCA1a* and *ZmCCA1b*. Expression level indicates fragments per kilobase pair of exon model per million fragments mapped (FPKM).
