# Supplementary material for: Temporal Shift of Circadian-Mediated Gene Expression and Carbon Fixation Contributes to Biomass Heterosis in Maize Hybrids
Source: PLoS Genet. 2016 Jul 28;12(7):e1006197. doi: 10.1371/journal.pgen.1006197 (PMC4965137; doi:10.1371/journal.pgen.1006197)
Supplement: S3 Fig — (A) A plot of period versus relative amplitude error (RAE) of CAB2:LUC activity in wild-type (Col-0), Vec-1, ZmCCA1a-OX7, ZmCCA1b-OX2 and CCA1-OX2 under LL (means ± SEM). RAE > 0.6 represents arrthymicity. Mean values were compared to those of the vector control line using Student’s t-test. Single and double asterisks indicate significant differences at p-value < 0.05 and p-value < 0.01, respectively. T2 plants were used in the analysis. (B) CAB2:LUC activity rhythms in wild-type (WS), cca1-11, CCA1:ZmCCA1a cca1-11, CCA1:ZmCCA1b cca1-11 and CCA1:CCA1 cca1-11 under LL (means ± SEM, n = 4–8). White and grey bars represent the subjective day and night, respectively. T1 plants were used in the analysis. T1 plants were used in the analysis. (C) A plot of period versus relative amplitude error (RAE) of CAB2:LUC activity in wild-type (WS), cca1-11, CCA1:ZmCCA1a cca1-11, CCA1:ZmCCA1b cca1-11 and CCA1:CCA1 cca1-11 under LL (means ± SEM). Mean values were compared to those of cca1-11 using Student’s t-test, *p-value < 0.05 and **p-value < 0.01. (PDF) [file pgen.1006197.s003.pdf]

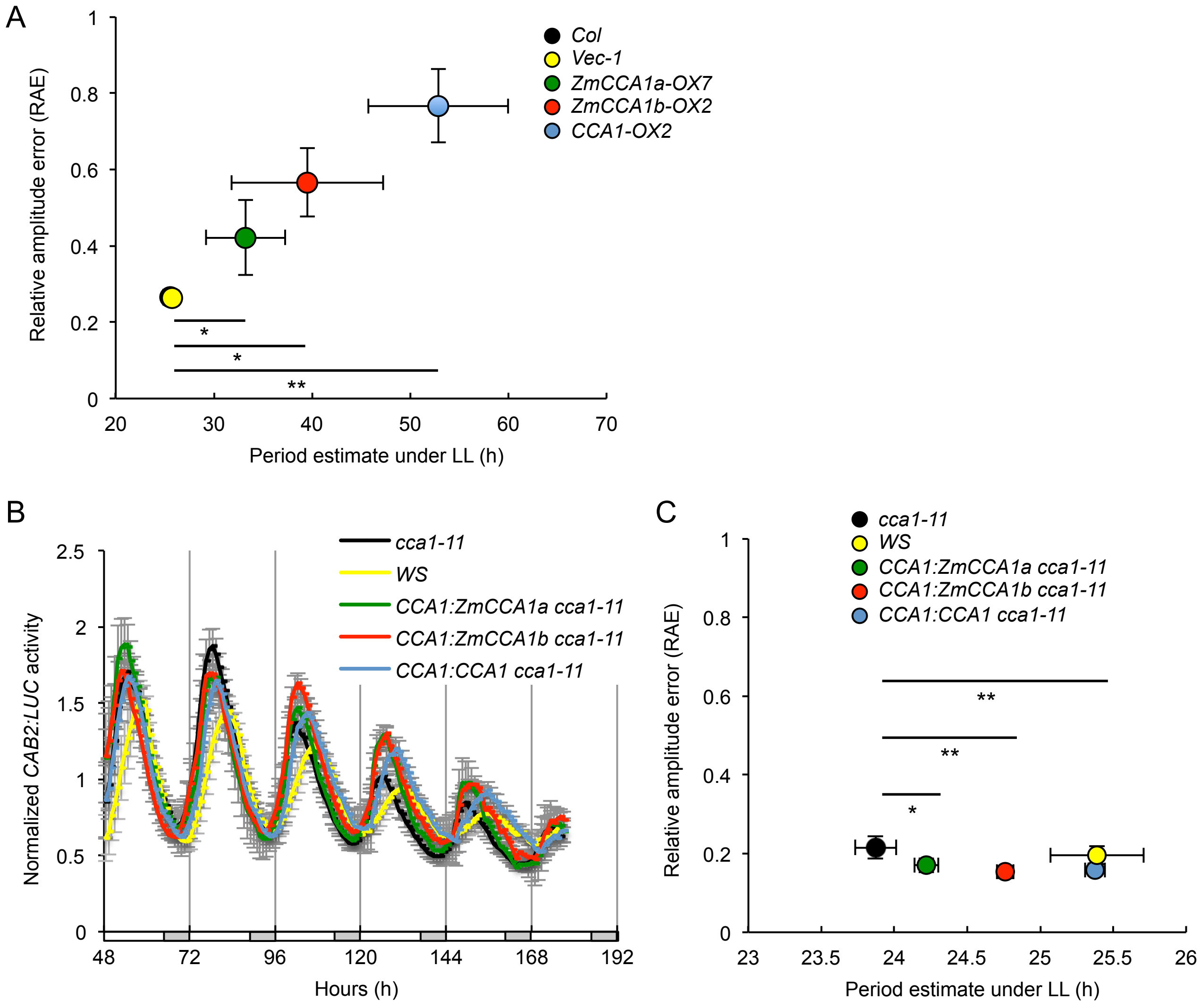

S3 Fig. Circadian characterization of maize CCA1 homologs in Arabidopsis. (A) A plot of period versus relative amplitude error (RAE) of CAB2:LUC activity in wild-type (Col-0), *Vec-1*, *ZmCCA1a-OX7*, *ZmCCA1b-OX2* and *CCA1-OX2* under constant light (LL) (means ± SEM). RAE > 0.6 represents arrthymicity. Mean values were compared to those of the vector control line using Student's t-test. Single and double asterisks indicate significant differences at p-value < 0.05 and p-value < 0.01, respectively. T2 plants were used in the analysis. (B) *CAB2*:*LUC* activity rhythms in wild-type (WS), *cca1-11*, *CCA1*:*ZmCCA1a cca1-11*, *CCA1*:*ZmCCA1b cca1-11* and *CCA1*:*CCA1 cca1-11* under LL (means ± SEM, n = 4-8). White and grey bars represent the subjective day and night, respectively. T1 plants were used in the analysis. T1 plants were used in the analysis. (C) A plot of period versus relative amplitude error (RAE) of *CAB2*:*LUC* activity in wild-type (WS), *cca1-11*, *CCA1*:*ZmCCA1a cca1-11*, *CCA1*:*ZmCCA1b cca1-11* and *CCA1*:*CCA1 cca1-11* under LL (means ± SEM). Mean values were compared to those of *cca1-11* using Student's t-test, *p-value < 0.05 and **p-value < 0.01.
