# Supplementary material for: Temporal Shift of Circadian-Mediated Gene Expression and Carbon Fixation Contributes to Biomass Heterosis in Maize Hybrids
Source: PLoS Genet. 2016 Jul 28;12(7):e1006197. doi: 10.1371/journal.pgen.1006197 (PMC4965137; doi:10.1371/journal.pgen.1006197)
Supplement: S4 Fig — (A) Immunodetection of ZmCCA1s in the maize inbreds and hybrids using the polyclonal antibodies used for our ChIP-seq. Histone H3 protein was used as a loading control. Plant tissues were collected at time points as labeled under the diurnal condition and used for protein extraction. (B) Workflow of computational analysis of ZmCCA1s ChIP-seq. Summary of mapping statistics is provided in S1 Table. (C) Line graph displays pairwise correlations between biological replicates calculated by Pearson correlation coefficients in windows of 1-kb over mapped read files. (PDF) [file pgen.1006197.s004.pdf]

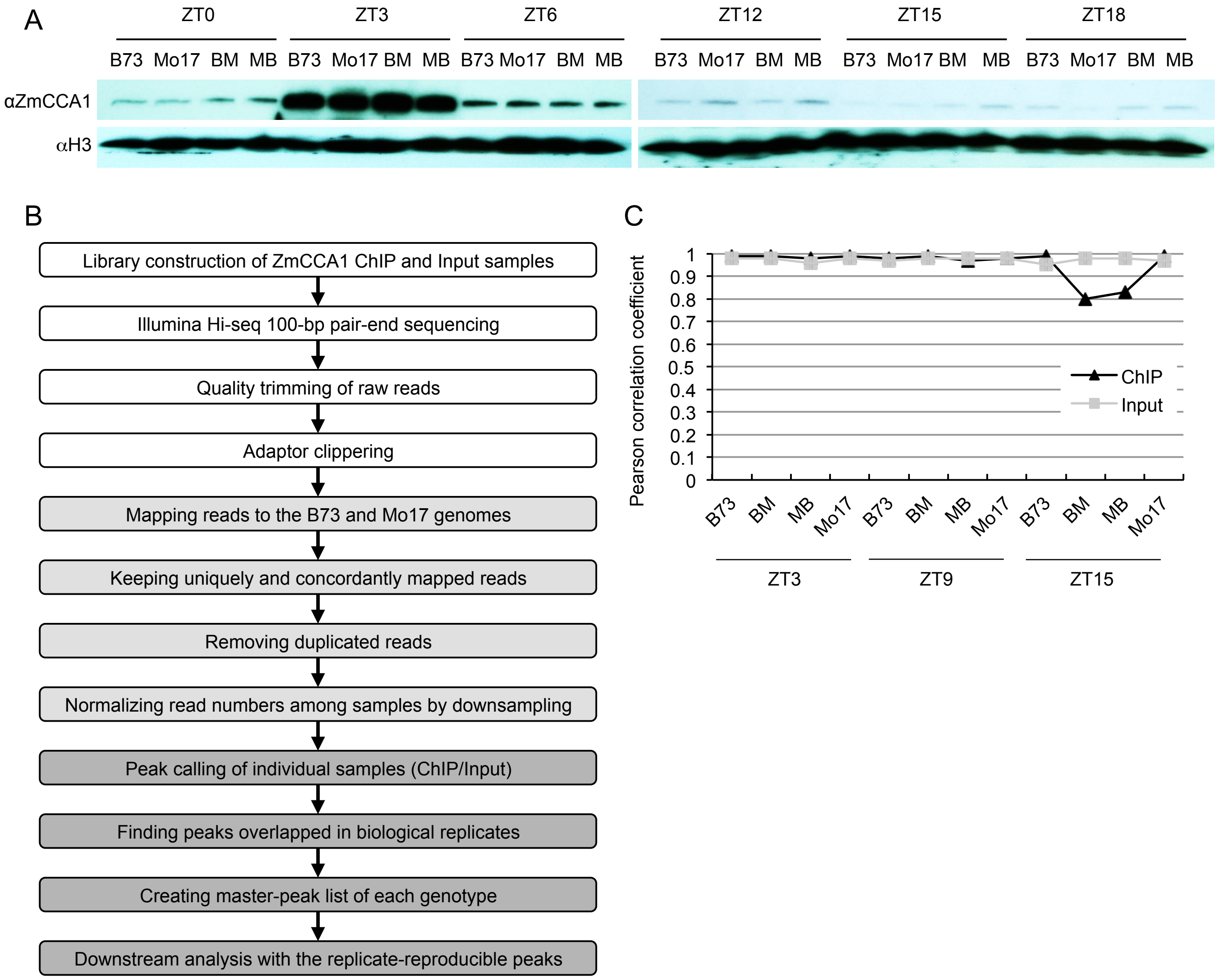

S4 Fig. Specificity of antibody against ZmCCA1s and computational pipeline for ChIP-seq analysis. (A) Immunodetection of ZmCCA1s in the maize inbreds and hybrids using the polyclonal antibodies used for our ChIP-seq. Histone H3 protein was used as a loading control. Plant tissues were collected at time points as labeled under the diurnal condition and used for protein extraction. (B) Workflow of computational analysis of ZmCCA1 ChIP-seq. Summary of mapping statistics is provided in S1 Table. (C) Line graph displays pairwise correlations between biological replicates calculated by Pearson correlation coefficients in windows of 1-kb over mapped read files.
