# Supplementary material for: Temporal Shift of Circadian-Mediated Gene Expression and Carbon Fixation Contributes to Biomass Heterosis in Maize Hybrids
Source: PLoS Genet. 2016 Jul 28;12(7):e1006197. doi: 10.1371/journal.pgen.1006197 (PMC4965137; doi:10.1371/journal.pgen.1006197)
Supplement: S5 Fig — (A) Histogram showing genomic annotation of ZmCCA1-binding peaks. Genome indicates the maize genome fraction as a control. Significant differences between Genome and each genotype are shown above each category using Fisher’s exact test. (B) Local enrichment of ZmCCA1-binding at putative maize clock genes at ZT3, ZT9 and ZT15. The Y-axis indicates input-subtracted read density on a same-scale for all genotypes and time-points. Arrows indicate gene orientation. (PDF) [file pgen.1006197.s005.pdf]

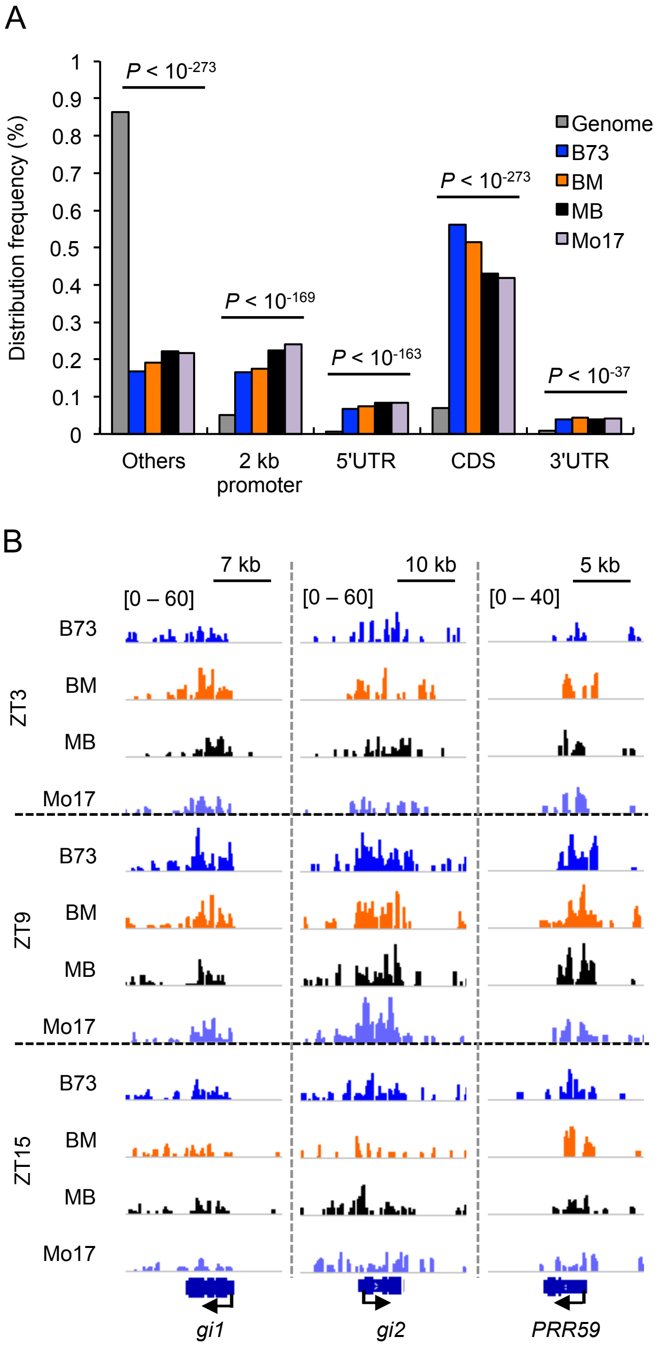

S5 Fig. Relative distribution of ZmCCA1-binding peaks across genomic regions and binding motifs found in the peaks. (A) Histogram showing genomic annotation of ZmCCA1-binding peaks. Genome indicates the maize genome fraction as a control. Significant differences between Genome and each genotype are shown above each category using Fisher's exact test. (B) Local enrichment of ZmCCA1-binding at putative maize clock genes at ZT3, ZT9 and ZT15. The Y-axis indicates input-subtracted read density on a same-scale for all genotypes and time-points. Arrows indicate gene orientation.
