# Supplementary material for: Temporal Shift of Circadian-Mediated Gene Expression and Carbon Fixation Contributes to Biomass Heterosis in Maize Hybrids
Source: PLoS Genet. 2016 Jul 28;12(7):e1006197. doi: 10.1371/journal.pgen.1006197 (PMC4965137; doi:10.1371/journal.pgen.1006197)
Supplement: S6 Fig — (A-B) Relative expression levels (means ± SEM, n = 3) of ZmTOC1a (A) and ZmPRR59 (B) every 3 hours in a 24 h period analyzed by qRT-PCR (light/dark cycle is shown below the histogram). Significant difference between hybrids and MPV was calculated using Student’s t-test, *p-value < 0.05 and **p-value < 0.01. The relative expression level in MPV at ZT0 was set to 1. (C) Hierarchically clustered heatmap showing the ChIP signals of ZmCCA1-binding (Z-scores) on ZmCCA1-binding peaks of each genotype. (D) Proportion of ZmCCA1-binding targets that were not shared. Statistical differences of ZT3 or ZT9 frequencies between the inbreds and F1 hybrids were calculated using Fisher’s exact test. Blue and purple values indicate the comparison with B73 and Mo17, respectively. The number of peaks or genes is shown above each bar. (E) Proportion of ZmCCA1-binding targets that were specific to either hybrids or inbreds. Statistical differences of ZT3, ZT9 or ZT15 frequencies between the hybrid-specific and inbred-specific target genes were calculated using Fisher’s exact test (ZT3, p-value = 2.2E-15; ZT9, p-value = 3.6E-15; ZT15, p-value = 2.9E-15). The number of genes is shown above each bar. (PDF) [file pgen.1006197.s006.pdf]

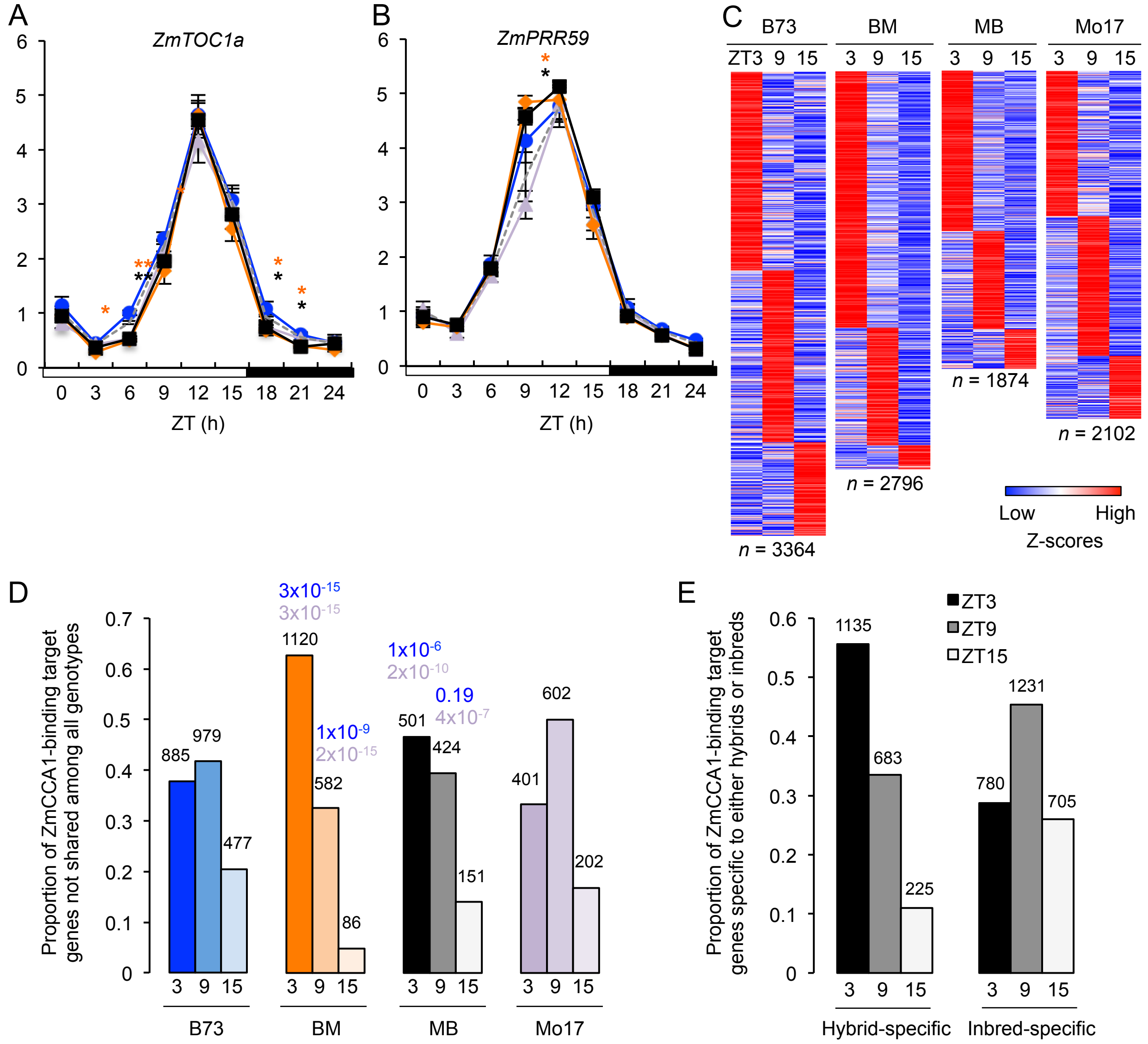

S6 Fig. Temporal regulation of ZmCCA1-binding targets and nonadditive expression of putative clock homolog genes. (A-B) Relative expression levels (means ± SEM, n = 3) of *ZmTOC1a* (A) and *ZmPRR59* (B) every 3 hours in a 24 h period analyzed by qRT-PCR (light/dark cycle is shown below the histogram). Significant difference between hybrids and MPV was calculated using Student's t-test, *p-value < 0.05 and **p-value < 0.01. The relative expression level in MPV at ZT0 was set to 1. (C) Hierarchically clustered heatmap showing the ChIP signals of ZmCCA1-binding (Z-scores) on ZmCCA1-binding peaks of each genotype. (D) Proportion of ZmCCA1-binding targets that were not shared. Statistical differences of ZT3 or ZT9 frequencies between the inbreds and F1 hybrids were calculated using Fisher's exact test. Blue and purple values indicate the comparison with B73 and Mo17, respectively. The number of peaks or genes is shown above each bar. (E) Proportion of ZmCCA1-binding targets that were specific to either hybrids or inbreds. Statistical differences of ZT3, ZT9 or ZT15 frequencies between the hybrid-specific and inbred-specific target genes were calculated using Fisher's exact test (ZT3, p-value = 2.2E-15; ZT9, p-value = 3.6E-15; ZT15, p-value = 2.9E-15). The number of genes is shown above each bar.
