# Supplementary material for: Temporal Shift of Circadian-Mediated Gene Expression and Carbon Fixation Contributes to Biomass Heterosis in Maize Hybrids
Source: PLoS Genet. 2016 Jul 28;12(7):e1006197. doi: 10.1371/journal.pgen.1006197 (PMC4965137; doi:10.1371/journal.pgen.1006197)
Supplement: S7 Fig — (A) Venn diagram showing genotype-dependent and -shared ZmCCA1s targets in the inbreds and hybrids (B73, n = 2521; Mo17, n = 1522; BM, n = 2063; MB, n = 1406). (B-D) Venn diagram showing genotype-dependent and -shared ZmCCA1s targets at ZT3 (B), ZT9 (C) or ZT15 (D). Inbred-specific or hybrid-specific genes are indicated by dashed circles. Enriched functional pathways in GO analysis are shown with false-discovery rate adjusted p-value (Hypergeometric test). Full lists of GO terms are available in S3 Table. (PDF) [file pgen.1006197.s007.pdf]

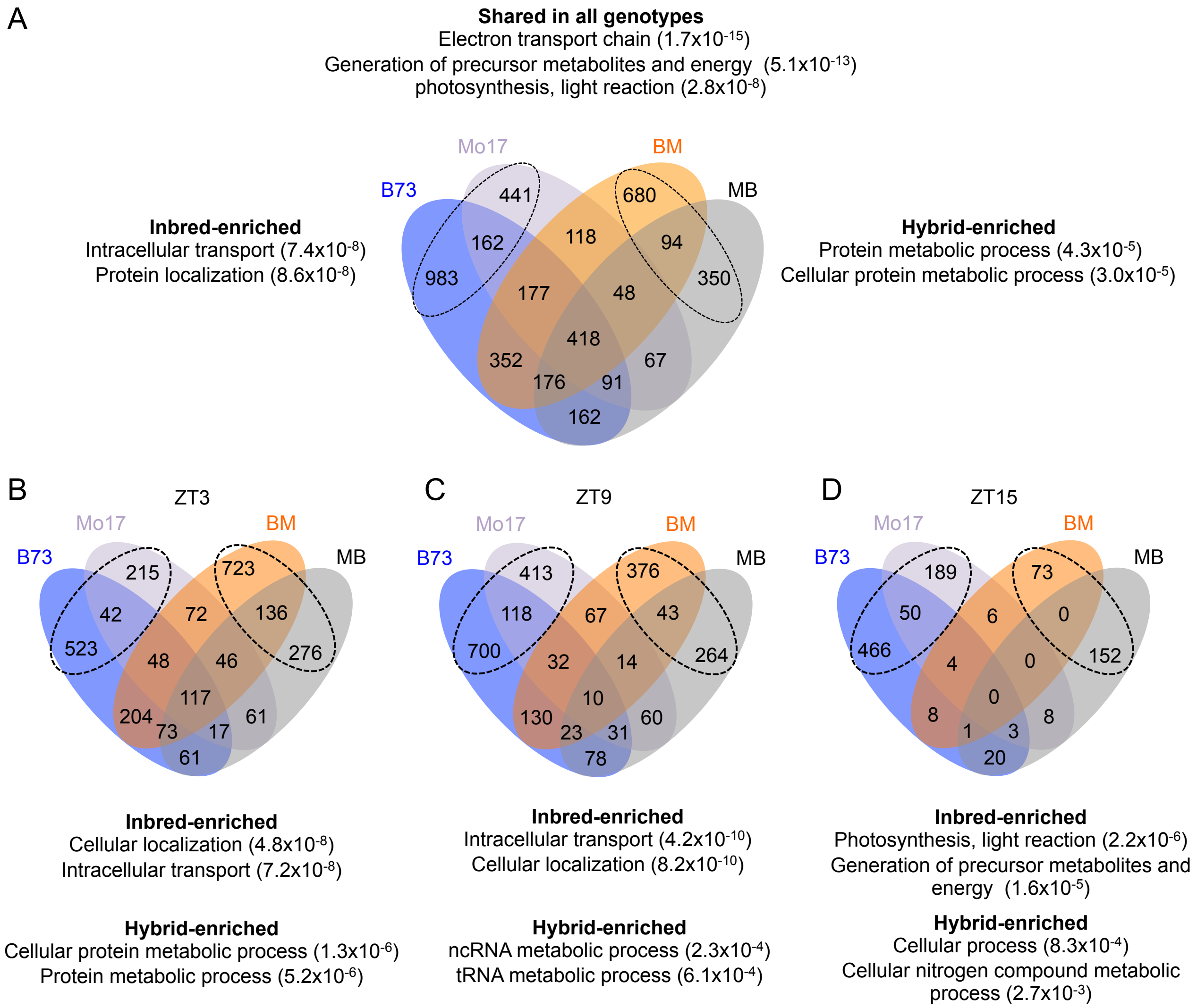

S7 Fig. Diverse biological pathways are enriched in ZmCCA1-binding target genes. (A) Venn diagram showing genotype-dependent and -shared ZmCCA1s targets in the inbreds and hybrids (B73, n = 2521; Mo17, n = 1522; BM, n = 2063; MB, n = 1406). (B-D) Venn diagram showing genotype-dependent and -shared ZmCCA1s targets at ZT3 (B), ZT9 (C) or ZT15 (D). Inbred-specific or hybrid-specific genes are indicated by dashed circles. Enriched functional pathways in GO analysis are shown with false-discovery rate adjusted p-value (Hypergeometric test). Full lists of GO terms are available in S3 Table.
