# Supplementary material for: Temporal Shift of Circadian-Mediated Gene Expression and Carbon Fixation Contributes to Biomass Heterosis in Maize Hybrids
Source: PLoS Genet. 2016 Jul 28;12(7):e1006197. doi: 10.1371/journal.pgen.1006197 (PMC4965137; doi:10.1371/journal.pgen.1006197)
Supplement: S8 Fig — (A-D) Relative expression levels (means ± SEM, n = 3) of ZM2G126988 (A), ZM2G427369 (B), ZM2G294732 (C), and ZM2G448142 (D) every 3 hours in a 24-hour period (light/dark cycle is shown below the histogram). The relative expression level in MPV at ZT0 was set to 1. Significant difference between MPV and hybrids was calculated using Student’s t-test, *p-value < 0.05 and **p-value < 0.01. The right panel for each gene shows ZmCCA1-binding peaks at ZT3, ZT9 and ZT15. The Y-axis indicates input-subtracted read density on a same-scale for all genotypes and time-points. Arrows indicate gene orientation. (E-F) ZmCCA1-binding enrichments of ZM2G398288 (E) and gi1 (F) at ZT3, ZT9 and ZT15. Notations are the same as above. (G) Heatmap of qRT-PCR data (|log2(F1/MPV)|, n = 3) showing nonadditive expression of maize CCA1 and carbon fixation genes in the hybrids at ZT3 except for ZM2G412611 at ZT9. CNR2 was used as a marker for nonadditive expression in the seedlings. (PDF) [file pgen.1006197.s008.pdf]

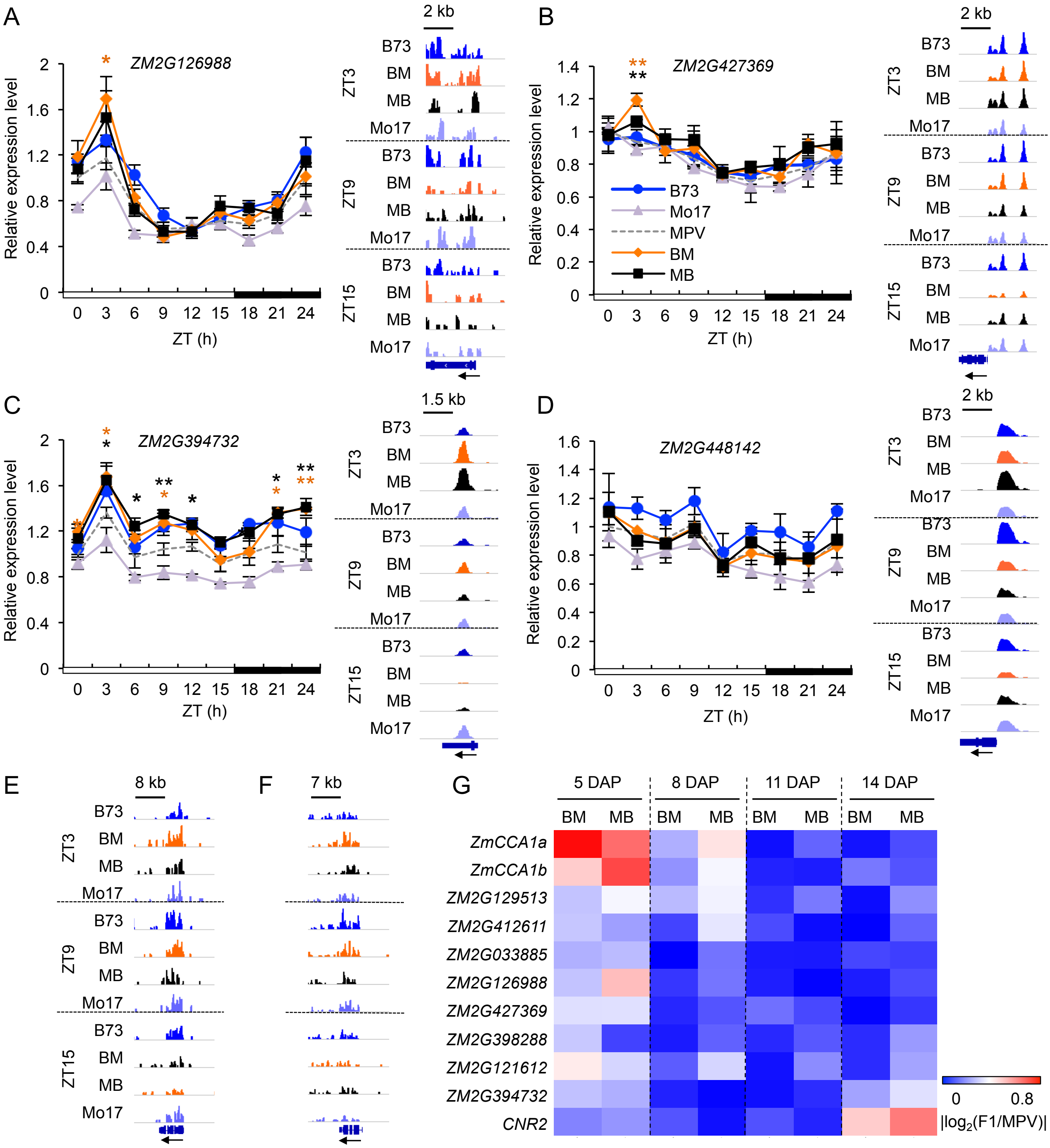

S8 Fig. Temporal shift of ZmCCA1-binding target (carbon fixation) genes and their expression. (A-D) Relative expression levels (means ± SEM, n = 3) of *ZM2G126988* (A), *ZM2G427369* (B), *ZM2G294732* (C), and *ZM2G448142* (D) every 3 hours in a 24-hour period (light/dark cycle is shown below the histogram). The relative expression level in MPV at ZT0 was set to 1. Significant difference between MPV and hybrids was calculated using Student's t-test, *p-value < 0.05 and **p-value < 0.01. The right panel for each gene shows ZmCCA1-binding peaks at ZT3, ZT9 and ZT15. The Y-axis indicates input-subtracted read density on a same-scale for all genotypes and time-points. Arrows indicate gene orientation. (E-F) ZmCCA1-binding enrichments of *ZM2G398288* (E) and *gi1* (F) at ZT3, ZT9 and ZT15. Notations are the same as above. (G) Heatmap of qRT-PCR data ($|\log_2(F1/MPV)|$, n = 3) showing nonadditive expression of maize *CCA1* homologous and carbon fixation genes in the hybrids at ZT3 except for *ZM2G412611* at ZT9. *CNR2* was used as a marker for nonadditive expression in the seedlings.
