# Supplementary material for: Temporal Shift of Circadian-Mediated Gene Expression and Carbon Fixation Contributes to Biomass Heterosis in Maize Hybrids
Source: PLoS Genet. 2016 Jul 28;12(7):e1006197. doi: 10.1371/journal.pgen.1006197 (PMC4965137; doi:10.1371/journal.pgen.1006197)
Supplement: S9 Fig — Binding of recombinant ZmCCA1b (rZmCCA1b) to the promoters of ZmCCA1-binding target genes associated with carbon fixation in vitro. Radioisotope-labeled DNA probes (endogenous promoter fragments) were incubated in the presence of MBP (1 pmol), rZmCCA1a (1 pmol), and rZmCCA1b (0.5, 1 and 2 pmol). Shifted protein-DNA complexes are indicated by the arrow. Competitors: 25X, 50X and 100X molar excess of unlabeled prompter DNA. M: DNA in which EE or CBS site was mutated; N: no EE or CBS site in the DNA fragment. Location of probes for each gene is shown below the gel image. Arrowheads represent EE or CBS site. Numbers are relative to the transcription start site (+1) and 5’ UTR (grey box). (PDF) [file pgen.1006197.s009.pdf]

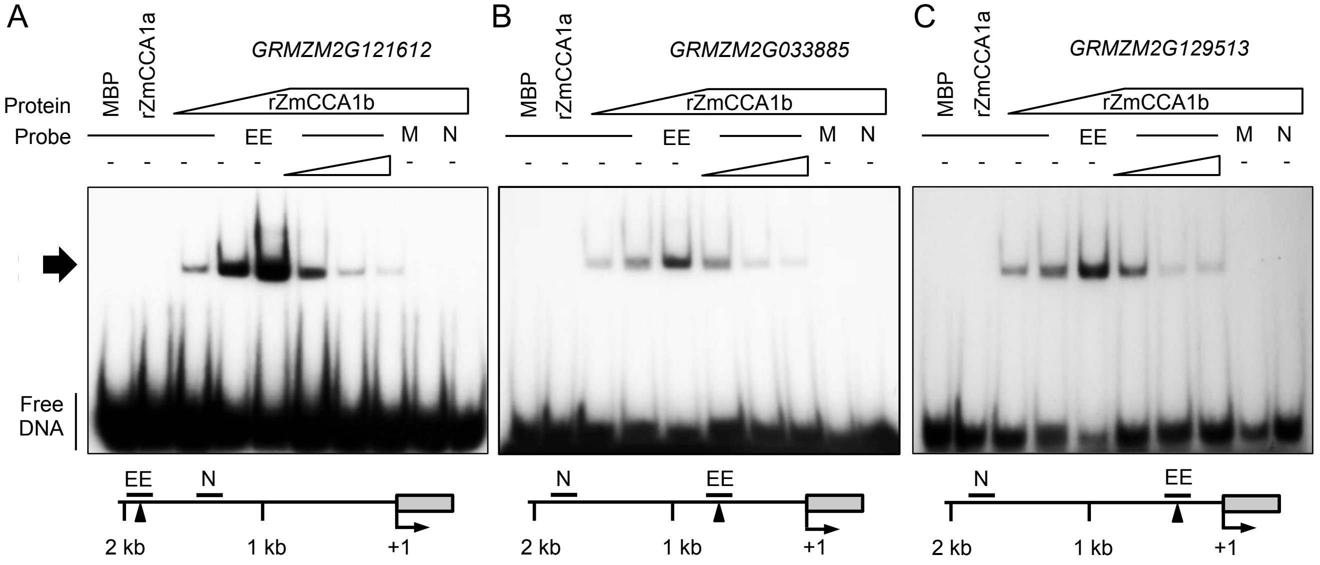

S9 Fig. Verification of ZmCCA1-binding to promoters of carbon fixation genes *in vitro*. Binding of recombinant ZmCCA1b (rZmCCA1b) to the promoters of ZmCCA1-binding target genes associated with carbon fixation *in vitro*. Radioisotope-labeled DNA probes (endogenous promoter fragments) were incubated in the presence of MBP (1 pmol), rZmCCA1a (1 pmol), and rZmCCA1b (0.5, 1 and 2 pmol). Shifted protein-DNA complexes are indicated by the arrow. Competitors: 25X, 50X and 100X molar excess of unlabeled prompter DNA. M: DNA in which EE or CBS site was mutated; N: no EE or CBS site in the DNA fragment. Location of probes for each gene is shown below the gel image. Arrowheads represent EE or CBS site. Numbers are relative to the transcription start site (+1) and 5' UTR (grey box).
