# Supplementary material for: Temporal Shift of Circadian-Mediated Gene Expression and Carbon Fixation Contributes to Biomass Heterosis in Maize Hybrids
Source: PLoS Genet. 2016 Jul 28;12(7):e1006197. doi: 10.1371/journal.pgen.1006197 (PMC4965137; doi:10.1371/journal.pgen.1006197)
Supplement: S1 Table — (DOCX) [file pgen.1006197.s010.docx]

**S1 Table. ChIP-seq mapping summary**

| **Genotype** | **ZT** | **Sample type** | **Biol. rep.** | **# Raw read-pairs** | **% Mapping rate** | **# Uniquely mapped* read-pairs (with duplicates)** | **# Uniquely mapped* read-pairs (without duplicates)** | **# Down-sampled uniquely mapped reads (without duplicates)** |
| --- | --- | --- | --- | --- | --- | --- | --- | --- |
|  |  |  |  |  |  |  |  |  |
|  |  |  |  |  |  |  |  |  |
|  |  |  |  |  |  |  |  |  |
| B73 | 3 | ChIP | 1 | 18,812,717 | 63.46 | 5,293,149 | 2,135,504 | 2,096,442 |
| BM | 3 | ChIP | 1 | 19,241,776 | 55.04 | 4,862,904 | 1,152,116 | 2,096,146 |
| MB | 3 | ChIP | 1 | 19,391,273 | 57.58 | 4,980,079 | 1,170,503 | 2,095,434 |
| Mo17 | 3 | ChIP | 1 | 21,937,289 | 61.83 | 5,601,530 | 2,015,033 | 2,097,538 |
| B73 | 3 | Input | 1 | 33,120,634 | 71.35 | 9,440,228 | 9,129,653 | 10,281,272 |
| BM | 3 | Input | 1 | 39,830,759 | 72.53 | 11,094,039 | 10,493,882 | 10,284,930 |
| MB | 3 | Input | 1 | 31,056,428 | 72.04 | 8,572,234 | 8,242,457 | 10,279,580 |
| Mo17 | 3 | Input | 1 | 38,226,163 | 71.24 | 9,761,317 | 9,662,472 | 10,273,896 |
| B73 | 3 | ChIP | 2 | 17,730,331 | 56.06 | 4,556,943 | 1,670,482 | 2,094,662 |
| BM | 3 | ChIP | 2 | 12,721,100 | 54.30 | 3,083,729 | 1,144,601 | 2,095,618 |
| MB | 3 | ChIP | 2 | 9,912,528 | 55.07 | 2,308,541 | 1,047,850 | 2,095,688 |
| Mo17 | 3 | ChIP | 2 | 16,002,796 | 62.29 | 4,194,671 | 1,838,532 | 2,094,916 |
| B73 | 3 | Input | 2 | 28,044,403 | 70.72 | 7,921,851 | 7,567,949 | 10,284,620 |
| BM | 3 | Input | 2 | 40,580,485 | 71.00 | 11,078,249 | 10,297,926 | 10,279,786 |
| MB | 3 | Input | 2 | 23,677,951 | 70.50 | 6,377,428 | 6,162,894 | 10,280,814 |
| Mo17 | 3 | Input | 2 | 25,817,966 | 70.28 | 6,547,885 | 6,426,413 | 10,281,472 |
| B73 | 9 | ChIP | 1 | 15,804,920 | 55.80 | 3,841,784 | 2,411,046 | 2,095,688 |
| BM | 9 | ChIP | 1 | 20,531,767 | 61.49 | 5,331,011 | 3,129,300 | 2,097,152 |
| MB | 9 | ChIP | 1 | 14,508,856 | 59.43 | 3,730,487 | 2,109,493 | 2,096,694 |
| Mo17 | 9 | ChIP | 1 | 16,072,514 | 62.01 | 4,126,349 | 2,537,406 | 2,094,548 |
| B73 | 9 | Input | 1 | 45,698,716 | 70.80 | 12,872,822 | 12,495,611 | 10,291,344 |
| BM | 9 | Input | 1 | 45,525,047 | 72.13 | 12,562,180 | 12,198,961 | 10,293,288 |
| MB | 9 | Input | 1 | 32,734,396 | 71.88 | 9,076,047 | 8,776,820 | 10,283,360 |
| Mo17 | 9 | Input | 1 | 22,502,176 | 70.02 | 5,730,267 | 5,600,457 | 10,282,640 |
| B73 | 9 | ChIP | 2 | 15,976,189 | 59.28 | 4,189,399 | 2,441,427 | 2,094,674 |
| BM | 9 | ChIP | 2 | 16,855,110 | 63.10 | 4,587,095 | 2,812,749 | 2,096,910 |
| MB | 9 | ChIP | 2 | 12,028,647 | 66.05 | 3,351,021 | 2,310,537 | 2,096,344 |
| Mo17 | 9 | ChIP | 2 | 10,590,660 | 57.10 | 2,554,970 | 1,354,164 | 2,095,864 |
| B73 | 9 | Input | 2 | 33,023,886 | 71.46 | 9,325,995 | 9,116,463 | 10,286,712 |
| BM | 9 | Input | 2 | 32,274,256 | 71.61 | 8,951,030 | 8,628,640 | 10,285,338 |
| MB | 9 | Input | 2 | 28,651,362 | 71.90 | 7,913,713 | 7,643,434 | 10,284,158 |
| Mo17 | 9 | Input | 2 | 23,882,879 | 70.22 | 6,005,211 | 5,877,309 | 10,287,290 |
| B73 | 15 | ChIP | 1 | 20,288,627 | 61.04 | 5,533,406 | 2,943,357 | 2,096,714 |
| BM | 15 | ChIP | 1 | 18,934,373 | 62.00 | 5,008,290 | 2,794,833 | 2,094,138 |
| MB | 15 | ChIP | 1 | 16,098,005 | 60.58 | 4,112,479 | 1,787,244 | 2,093,534 |
| Mo17 | 15 | ChIP | 1 | 13,538,518 | 48.88 | 2,661,517 | 1,772,091 | 2,096,864 |
| B73 | 15 | Input | 1 | 26,295,719 | 71.00 | 7,443,824 | 7,113,794 | 10,281,406 |
| BM | 15 | Input | 1 | 41,621,018 | 71.67 | 11,300,917 | 10,795,524 | 10,282,566 |
| MB | 15 | Input | 1 | 33,011,407 | 71.82 | 9,039,475 | 8,832,426 | 10,285,672 |
| Mo17 | 15 | Input | 1 | 21,580,961 | 69.55 | 5,305,022 | 5,141,882 | 10,283,752 |
| B73 | 15 | ChIP | 2 | 21,885,820 | 62.58 | 5,867,350 | 2,930,744 | 2,096,026 |
| BM | 15 | ChIP | 2 | 25,624,124 | 69.35 | 6,859,825 | 5,219,660 | 2,096,520 |
| MB | 15 | ChIP | 2 | 17,109,424 | 67.06 | 4,578,249 | 3,292,002 | 2,097,478 |
| Mo17 | 15 | ChIP | 2 | 14,911,933 | 62.09 | 3,700,185 | 2,054,424 | 2,096,774 |
| B73 | 15 | Input | 2 | 28,337,553 | 71.46 | 7,865,577 | 7,590,052 | 10,286,728 |
| BM | 15 | Input | 2 | 39,869,030 | 72.33 | 10,849,595 | 10,545,382 | 10,281,684 |
| MB | 15 | Input | 2 | 32,179,907 | 72.69 | 8,745,697 | 8,487,720 | 10,279,704 |
| Mo17 | 15 | Input | 2 | 37,958,599 | 71.16 | 9,569,442 | 9,242,678 | 10,285,712 |

*“Uniquely mapped reads” means concordantly paired-reads mapped to the genome exactly 1 time in this study.
