# Supplementary material for: Temporal Shift of Circadian-Mediated Gene Expression and Carbon Fixation Contributes to Biomass Heterosis in Maize Hybrids
Source: PLoS Genet. 2016 Jul 28;12(7):e1006197. doi: 10.1371/journal.pgen.1006197 (PMC4965137; doi:10.1371/journal.pgen.1006197)
Supplement: S4 Table — (DOCX) [file pgen.1006197.s013.docx]

**S4 Table. List of primers used in qRT-PCR and ChIP-qPCR.**

| **qRT-PCR analysis (5' to 3')** | |  | |  |
| --- | --- | --- | --- | --- |
| **Name** | **Gene ID** | | **Forward sequence** | **Reverse sequence** |
| *ZmCCA1a* | GRMZM2G474769 | | TTGGTGAGCCAAGGGCTTCCTTT | CTGTTCGGCGCAATTTCAGCTT |
| *ZmCCA1b* | *GRMZM2G014902* | | CGAAGCATCCACATAATTGATTT | GCACTGCATTGCAAGATCTGA |
| *gi1* | *GRMZM2G107101* | | GCAATACCAGCTCATTGGATAGTGT | CAACATCGCCATTCAGTAGGACCTC |
| *ZmPRR59* | *GRMZM2G135446* | | TCTTGCTGCCTCCCAATGACCATA | ACTACTTGCACCAGCATCTTCCCT |
| *ZmTOC1a* | *GRMZM2G020081* | | GCCAACCAATACGGATGTCAT | TGGATCGTCATCTTCGTCTTCA |
| *-* | *GRMZM2G398288* | | TCCAATGATGCAGTGGTTGGTA | AGCTGGCTTTGAAGGTGAATCT |
| *-* | *GRMZM2G427369* | | TGGTCCACAGGGTTCATAACTA | AACAGTCGGACAAGTGGGTAAT |
| *-* | *GRMZM2G126988* | | TAAGTGTCGCAAGGGTGGATGACA | TGGTACGAAGGGTTTCGTTGGTGA |
| *-* | *GRMZM2G129513* | | CGTGTTCAGCATGCCATGCAGATC | TCTCAGCAAGCAATTCAGCTTCGC |
| *-* | *GRMZM2G394732* | | GCATTAATTGCAACTTCCTCAG | CCAGAAAGACTAATCCAATCCA |
| *-* | *GRMZM2G412611* | | TGTGGCAAAGAGTGTCAAGGGAGA | TTCCTTGCTCGGACTGACACATGA |
| *-* | *GRMZM2G448142* | | TCTCGACAATACGAAGCAATAGG | TGCTTAGTGTGTGACTCGTTAG |
| *CNR2* | *GRMZM2G151230* | | CGGCGGCGGCGGCTACTACCAG | GGCAGTCGTCGAAGCAGTTGCAGA |
| *18srRNA* | *AF168884* | | TCTGTGATGCCCTTAGATGTTCTG | CTGTCGGCCAAGGCTATATACT |
| **ChIP-qPCR analysis (5' to 3')** | | |  |  |
| **Name** | **Gene ID** | | **Forward sequence** | **Reverse sequence** |
| *gi1* | *GRMZM2G107101* | | GAACTTCCATTTCAGCCATCC | GAAATAATCAAGGTGACGGG |
| *gi2* | *GRMZM5G844173* | | CAGTGTTGATTACAGGTGCTC | GATTCTGACATGCTTGGTAGC |
| *-* | *GRMZM2G033885* | | GGCAACCATGAAAACAATTGGTG | CCGGACTCACGAAGGTGAATGCA |
| *-* | *GRMZM2G129513* | | GAGCACAGATCCCTTGGTAT | CATCCTAGCATTGATATCGT |
| *-* | *GRMZM2G121612* | | GGTAGCCTGAAATCCTTATGCC | GAGCAACTAAACTCCAAAGATC |
| *-* | *GRMZM2G337113* | | CAACCAAACATACAATCTATCTC | GCTGATATATTCACCAAACCTC |
