# Supplementary material for: Temporal Shift of Circadian-Mediated Gene Expression and Carbon Fixation Contributes to Biomass Heterosis in Maize Hybrids
Source: PLoS Genet. 2016 Jul 28;12(7):e1006197. doi: 10.1371/journal.pgen.1006197 (PMC4965137; doi:10.1371/journal.pgen.1006197)
Supplement: S5 Table — (DOCX) [file pgen.1006197.s014.docx]

**S5 Table. List of oligonucleotides used in EMSA.**

| **Name** | **Gene ID** | **Description** | **Sequence** |
| --- | --- | --- | --- |
| *gi1* | *GRMZM2G107101* | F | GCATCGCGGAAAAAATCTTTTTCTCGCC |
|  |  | R | GGCGAGAAAAAGATTTTTTCCGCGATGC |
|  |  | M_F | GCATCGCGGAAAAAAgagTTTTCTCGCC |
|  |  | M_R | GGCGAGAAAActcTTTTTTCCGCGATGC |
|  |  | N_F | GTCCGAGGCTAGGCTCGGGTGAAGCGTG |
|  |  | N_R | CACGCTTCACCCGAGCCTAGCCTCGGAC |
| *ZmPRR59* | *GRMZM2G135446* | F | CTCTTTAAAAAAAAATCTTCATTTAAGG |
|  |  | R | CCTTAAATGAAGATTTTTTTTTAAAGAG |
|  |  | M_F | CTCTTTAAAAAAAAAgagTCATTTAAGG |
|  |  | M_R | CCTTAAATGActcTTTTTTTTTAAAGAG |
|  |  | N_F | GGGTCAGGCCGTCACGGGTCCAATGGAC |
|  |  | N_R | GTCCATTGGACCCGTGACGGCCTGACCC |
| *ZmTOC1a* | *GRMZM2G020081* | F | GGCGAGTTTTAGATATTTTCACCCGCTCC |
|  |  | R | GGAGCGGGTGAAAATATCTAAAACTCGCC |
|  |  | M_F | GGCGAGTTTTccccgcgaTCACCCGCTCC |
|  |  | M_R | GGAGCGGGTGAtcgcggggAAAACTCGCC |
|  |  | N_F | GACCCCACCTAGCCGGTCTGTGTCTGTCC |
|  |  | N_R | GGACAGACACAGACCGGCTAGGTGGGGTC |
| - | *GRMZM2G033885* | F | TCAGCACTGAAAAATATCTGAGGTTGAAA |
|  |  | R | TTTCAACCTCAGATATTTTTCAGTGCTGA |
|  |  | M_F | TCAGCACTGAccccgcgaTGAGGTTGAAA |
|  |  | M_R | TTTCAACCTCAtcgcggggTCAGTGCTGA |
|  |  | N_F | AGGTAGTGCTTGGTATTGACATCAGACTT |
|  |  | N_R | AAGTCTGATGTCAATACCAAGCACTACCT |
| - | *GRMZM2G129513* | F | ATTAAATATAAAAATATCTAAATTCATAA |
|  |  | R | TTATGAATTTAGATATTTTTATATTTAAT |
|  |  | M_F | ATTAAATATAccccgcgaTAAATTCATAA |
|  |  | M_R | TTATGAATTTAtcgcggggTATATTTAAT |
|  |  | N_F | CGCGGTGAAGCATCACTGGGCCCAAGGGG |
|  |  | N_R | CCCCTTGGGCCCAGTGATGCTTCACCGCG |

EE or CBS elements are underlined. Mutated sequences are lower cases. F: forward strand; R: reverse strand; M: DNA in which EE or CBS site was mutated; N: no EE or CBS site in the DNA fragment.
